# Maturation of the medaka immune system depends on reciprocal interactions between the microbiota and the intestinal tract

**DOI:** 10.3389/fimmu.2023.1259519

**Published:** 2023-09-12

**Authors:** Hiyori Sakaguchi, Yuna Sato, Ryo Matsumoto, Joe Gomikawa, Namie Yoshida, Tomohiro Suzuki, Masaru Matsuda, Norimasa Iwanami

**Affiliations:** Center for Bioscience Research and Education, Utsunomiya University, Utsunomiya, Japan

**Keywords:** medaka, microbiota, immune system, germ-free, SCID

## Abstract

The interactions between the host immune system and intestinal microorganisms have been studied in many animals, including fish. However, a detailed analysis has not been performed in medaka, an established fish model for biological studies. Here, we investigated the effect of immunodeficiency on the microbiota composition and the effect of gut bacteria on intestinal epithelial development and immune responses in medaka. Chronological analysis of the intestinal microbiota of *interleukin 2 receptor subunit gamma (il2rg)* mutant medaka showed a gradual decrease in the evenness of operational taxonomic units, mainly caused by the increased abundance of the Aeromonadaceae family. Exposure of wild-type medaka to high doses of an intestine-derived opportunistic bacterium of the Aeromonadaceae family induced an inflammatory response, suggesting a harmful effect on adult *il2rg* mutants. In addition, we established germ-free conditions in larval medaka and observed large absorptive vacuoles in intestinal epithelial cells, indicating a block in epithelial maturation. Transcriptome analysis revealed a decrease in the expression of genes involved in the defense response, including the antimicrobial peptide gene *hepcidin*, whose expression is induced by lipopolysaccharide stimulation in normal larvae. These results show that reciprocal interactions between the microbiome and the intestinal tract are required for the maturation of the medaka immune system.

## 1 Introduction

The intestine develops initially under sterile conditions and is colonized by microbial communities after birth. The intestinal microbiome affects many aspects of host physiology, including nutrient uptake, energy homeostasis, adaptive immune homeostasis and control of inflammatory signaling (1–4). The gut microbiome plays a pivotal role in the initiation and progression of several diseases, including cancer (5).

Immune-compromised humans, including those with severe combined immunodeficiency (SCID), are known to have disrupted microbiota (6, 7). Defects in *INTERLEUKIN 2 RECEPTOR SUBUNIT GAMMA* (*IL2RG)* that encodes common γ chain (γc), a type I cytokine receptor, on the X chromosome, cause X-linked immunodeficiency (X-SCID) in humans (8). In zebrafish, a major genetic model fish, it has been shown that gut microbiota composition plays a crucial role in the maintenance of innate immunity (9). Furthermore, a recent study reported that *recombination-activating gene 1* (*rag1*) mutants lacking T and B cells and *il2rg* mutants, lacking T and natural killer (NK) cells, had a differential intestinal bacterial composition (10, 11); however, these studies did not analyze the changes in gut microbiota over time in detail.

Furthermore, the effect of commensal microbiota on the host immune response has been actively studied in zebrafish (12), largely by establishing protocols for growing fish under germ-free and gnotobiotic conditions (13, 14). Previous studies using a zebrafish germ-free model have demonstrated that colonization by commensals in newly hatched zebrafish primes neutrophils and induces several genes encoding proinflammatory and antiviral mediators, increasing the resistance of larvae to viral infection; commensal microbe recognition was found to be mediated mainly through a TLR/MyD88 signaling pathway, and professional phagocytes were identified as the source of these immune mediators (15). Moreover, endogenous microbiota establish the normal homeostatic level of neutrophils in the intestine; this process is mediated by lipopolysaccharide (LPS)-induced intestinal alkaline phosphatase through dephosphorylation of LPS and negative feedback of TLR signaling (16). The process of hatching itself changes the phenotype of skin keratinocytes that are responsible for both sensing the hyposmolarity of the aquatic environment and mediating immune effector mechanisms. The transition from the chorion environment to the rearing media is sensed through a transient potential receptor vanilloid 4/Ca^2+^/TGF-β–activated kinase 1/NF-κB signaling pathway (17). In addition, some studies have clarified that microbial signaling via Myd88 is important for intestinal epithelial cell proliferation, changes in the intestinal leukocyte subsets, and transcription of antibacterial genes (18, 19).

Medaka are freshwater fishes that have been established as model organisms for studies on genetics, developmental biology, and evolutionary biology (20). Inbred medaka strains are beneficial in physiological studies, especially those related to immune responses. Medaka shares genes involved in hematopoiesis and lymphocyte development with zebrafish and mammals (21–24). Changes in gut microbiota composition in immunocompromised medaka, including the inflammatory cytokine *interleukin 17 A/F1* mutant (25), and the effects of defects in its receptor *interleukin-17 receptor A1* on the downregulation of intestinal metabolism-related genes have been reported (26). However, the effects of defective adaptive immunity of medaka have not yet been studied. Furthermore, although *il2* and *il2ra* have not yet been identified in medaka, the medaka ortholog of *il2rg* is expected to be necessary for lymphocyte development, as reported in zebrafish (11, 27). In addition, the effects of germ-free conditions on intestinal epithelial development and immune function in medaka have not been studied.

Here, we established *il2rg* mutant medaka and identified dynamic changes in intestinal bacterial composition using chronological microbiota analysis. Furthermore, we established a germ-free condition for medaka and found the contribution of intestinal bacteria to intestinal epithelium development and the expression of genes in the defense response pathway. These findings indicate the requirement of reciprocal interactions between the microbiome and the intestinal tract for the maturation of the medaka immune system.

## 2 Materials and methods

### 2.1 Fish lines

The medaka (*Oryzias latipes*) lines were obtained from the National Bioresource Project (NBRP) Medaka and maintained at Utsunomiya University: inbred strain Hd-rR (strain ID: IB178), standard line OK-cab (strain ID: MT830), and cab-Tg (*rag1:egfp*), a transgenic medaka expressing *egfp* under the control of an immature lymphocyte-specific *rag1* promoter (strain ID: TG848) (22). Fish were maintained in temperature-controlled tanks (26°C) with a water circulation system under a 14 h light/10 h dark cycle. This study was conducted in accordance with the ethical guidelines of the Utsunomiya University Animal Experimentation Committee, and the experimental protocols were approved by the committee (approval no. A20c-0012). The developmental stages of medaka were designated as previously described (28).

### 2.2 Genome editing

Alt-R CRISPR-Cas9 crRNA targeting exon 3 of *il2rg* (ENSORLG00000001795), Alt-R CRISPR-Cas9 tracrRNA, and the Cas9 protein were purchased from Integrated DNA Technologies (IDT; Singapore). The sgRNA sequences are listed in **Table S1**. RNA–protein complexes were prepared according to the manufacturer’s instructions. Glass capillaries with 1.0 mm outer diameter were pulled (temperature 62°C, force: two light and two heavy weights provided by the manufacturer) using a micropipette puller PC-10 (Narishige Instruments, Tokyo, Japan). The capillary was filled with crRNA (0.75 mM), tracrRNA (1.5 mM), and 0.25 mg/mL Cas9 protein. Then 1–2 nL of the mixture was injected into 1-cell Cab embryos using a FemtoJet 4i (Eppendorf, Hamburg, Germany). G0 fish were crossed with wild-type (WT) Cab fish to establish the *il2rg* mutant strains. *il2rg^del4^* mutation was chosen for phenotypic analysis. The primers used for genotyping are shown in **Table S1**.

### 2.3 Imaging of medaka specimens

The larvae and adult medaka were anesthetized with 0.1% (v/v) 2-phenoxyethanol and immobilized in 3% methylcellulose. Images were captured using a DP73 digital camera (Olympus, Tokyo, Japan) under an MZ16F fluorescence stereo microscope (Leica, Houston, TX, USA).

### 2.4 Reverse transcription-PCR

RNA was extracted using the NucleoSpin RNA kit (Macherey-Nagel, Düren, Germany) with on-column DNase treatment. The RNA was reverse transcribed using a QuantiTect Reverse Transcription Kit (Qiagen). To detect *immunoglobulin Mu (igm)* and *t-cell receptor beta chain (tcrb)* cDNA after VDJ recombination, nested PCR was performed using primer sets targeting the variable (V) and constant (C) regions. Two V regions highly expressed in wild-type kidneys were chosen for RT-PCR analysis, respectively (data not shown). Amplification was performed in a thermal cycler using the following program: 1 min at 95°C, cycles of 10 sec at 95°C, 30 sec at 59°C, 30 sec at 72°C, and 5 min at 72°C. The cycle numbers were as follows: *actb* (*actin beta*), 25 cycles; *igm* (igV_H_-Cm) and *tcrb* (tcrVb-Cb2), 30 cycles plus nested 30 cycles. The primers used for RT-PCR are listed in **Table S1**.

Quantitative PCR (qPCR) was performed using THUNDERBIRD SYBR qPCR Mix (Toyobo, Osaka, Japan) and LightCycler 480 (Roche, Basel, Switzerland). The primers used for qPCR are listed in **Table S1**.

### 2.5 Metagenome analysis

The WT and *il2rg* mutants derived from incrosses of heterozygous *il2rg* mutants were raised together. After genotyping, they were maintained in a water tank separated by a mesh to share the same water environment. Intestinal bacterial DNA was extracted using a QuickGene DNA Tissue Kit (Kurabo, Osaka, Japan) following the method described by Wong et al. (29) with some modifications. Intestines were harvested from WT and *il2rg^-/-^* medaka using sterile tweezers, transferred to microfuge tubes with 0.8 g zirconia beads and 200 μL Tissue lysis Buffer MDT, and immediately frozen in liquid nitrogen. Intestines were then quickly thawed at 65°C, 50 μL lysozyme (50 mg/mL) was added, and incubated at 30°C for 45 min. Subsequently, the bacterial lysates were processed following the protocol of Bacterial Genomic DNA Extraction from Stool (DF-1) of the QuickGene DNA tissue kit (Kurabo).

The V3 and V4 regions of the 16S rRNA gene were amplified using primer pairs with Illumina overhang sequences (**Table S1**). Index tag sequences were added to the amplicons using the Nextera XT Index Kit v2 (Illumina) and sequenced using the MiSeq Reagent Kit v3 (Illumina). The 301-bp paired-end reads were trimmed to remove low-quality ends (quality score < 15) and adapters and merged using the DADA2 plugin of QIIME2 (https://qiime2.org/). The curated sequences were annotated using the QIIME2 q2-feature-classifier plugin and Silva 132 99% OTUs (full-length, seven-level taxonomy) classifier pre-trained to the full-length Silva database to assign taxonomy to all ribosomal sequence variants.

Alpha- and beta-diversity analyses were performed using the q2-diversity plugin wrapped in QIIME2 at a sampling depth of 15,000 sequences. Alpha diversity metrics (Shannon and Chao1 indices) were calculated, and statistical significance was determined using the Kruskal–Wallis test. For Beta diversity analysis, principal coordinate analysis (PCoA) was performed using Bray–Curtis and weighted UniFrac metrics and visualized using a QIIME2 emperor visualizer (ver. 2022.2.0). The statistical significances of weighted UniFrac metrics distances were calculated using Permutational Multivariate Analysis Of Variance (PERMANOVA).

Data were obtained from 2-3 independent experiments (**Table S2**).

### 2.6 Isolation and identification of adult gut-derived bacteria

The intestinal tracts were harvested from adult Hd-rR strain using sterile tweezers. After washing the surface of the intestine with sterile water, it was crushed in sterile phosphate-buffered saline (PBS) using a Power Masher II (Nippi Incorporated, Tokyo, Japan). Diluted intestinal bacteria was aerobically cultured on solid LB media at 28°C overnight, and the colonies were picked up for liquid culture. Bacterial DNA was extracted as described by Wong et al. (29) with some modifications. The bacterial pellet was suspended in 200 μL enzymatic lysis buffer (20 mM Tris-Cl (pH 8.0), 2 mM EDTA (pH 8.0), and 1% Triton X-100) and immediately frozen in liquid nitrogen. Subsequently, the samples were quickly thawed at 65°C, 20 μL 200 mg/mL lysozyme was added, and incubated at 30°C for 45 min. Next, 10 μL proteinase K (10 mg/mL) and 230 μL 40% guanidine hydrochloride were added, and the sample was incubated at 56°C for 30 min. Bacterial lysates were processed using a Mag Extractor-Genome (Toyobo) for DNA extraction.

The bacterial 16S rRNA gene was amplified using primers (16SrRNA 27F and 16SrRNA 1491R). Amplification was performed in a thermal cycler using the following program: 1 min at 95°C, 40 cycles of 10 sec at 95°C, 30 sec at 59°C, 30 sec at 72°C, and 5 min at 72°C. The amplicons were directly sequenced using 16SrRNA 27F, 16SrRNA 1491R and 16SrRNA 907R primers with BigDye Terminator v3.1Cyle Sequencing Kit (Thermo Fisher Scientific, Waltham, MA, USA) and analyzed using 3500 Genetic Analyzer (Applied Biosystems, Foster City, CA, USA). The primer sequences are listed in **Table S1**. Bacterial families were determined using the National Center for Biotechnology Information (NCBI) BLAST program (www.ncbi.nlm.nih.gov/cgi-bin/blast). A phylogenetic tree was generated with MegAlign (Lasergene, DNASTAR, Madison, WI) using MUSCLE alignment and a maximum likelihood model with 1000 bootstraps.

### 2.7 Bacterial exposure

One of the type A clones of *Aeromonas* isolated from the intestine of an adult Hd-rR strain was spread on a solid Brain Heart Infusion (BHI) medium (BD, Franklin Lakes, NJ). A single colony was picked and cultured in liquid BHI medium at 25°C overnight, then 1:100 dilution was further cultured at 25°C for 4 h until the growth phase was reached. Subsequently, the culture was centrifuged at 2000 × *g* for 10 min, and the supernatant was replaced with PBS. The OD600 of the culture was measured using an Od meter (Thomas Scientific, Swedesboro, NJ, USA). Two months post-fertilization (mpf), WT medaka were exposed to the bacteria at 6.5×10^7^ colony forming units (CFU)/mL in filtered water using the bath method. CFU of the bacteria were calculated as the colony numbers of diluted bacteria after overnight culture on the solid BHI medium. Twenty percent of the water-containing bacteria was replaced with filtered water every day.

### 2.8 Flow cytometry

Whole kidney marrow and spleen cells were prepared as previously described (30) with some modifications. Cells were obtained by pipetting the organs into 1 mL ice-cold 1% fetal bovine serum (FBS) in 0.9× PBS. After centrifugation, the pellet was gently mixed with 1 mL distilled water at 20°C by pipetting 4 times to lyse the erythrocytes by osmotic shock to prepare splenocytes, and immediately 1 mL of 1.8× PBS was added. Cells were washed with 1% FBS in 0.9× PBS by centrifugation and then filtered using a 40 μm-stainless mesh. Flow cytometry (FCM) was performed using a FACSLyric flow cytometer (BD Biosciences). Data analysis was performed using Flowjo10.6.2 (BD Biosciences).

### 2.9 Preparation of *Aeromonas* for gnotobiotic culture

A colony of *Aeromonas* isolated from the intestine of an adult Hd-rR strain was picked and transferred to liquid LB medium. OD600 values were measured using a UV-Vis Spectrophotometer V-630 (Jasco, Tokyo, Japan). By counting colony numbers after overnight culture on LB agar plate, CFU per OD600 were calculated so that 1× 10^6^ CFU/mL of *Aeromonas* culture was added to larvae.

### 2.10 Establishment of germ-free medaka larvae

Germ-free medaka were produced following the method used to produce germ-free zebrafish described by Melancon et al. (14) and Pham et al. (13) with some modifications. Eggs of Hd-rR strain were cultured in non-sterile embryonic culture medium (ECM) comprising 0.1% (w/v) NaCl, 0.003% (w/v) KCl, 0.004% (w/v) CaCl_2_-2H_2_O, 0.016% (w/v) MgSO_4_-7H_2_O at 28°C for 6 days.

At 6 days post-fertilization (dpf) (stage 36), embryos were transferred into sterile antibiotics-embryonic culture medium (AB-ECM) (200 μg/mL ampicillin, 5 μg/mL kanamycin, 0.5 μg/mL amphotericin B in ECM, filtered using 0.22 μm filters) and cultured at 28°C for 4 h. The embryos were then transferred to a sterile beaker containing AB-ECM. After washing three times with sterile ECM, embryos were soaked in 0.1% PVP-I solution (0.1% PVP-I in ECM, filtered using a 0.22 μm filter) for 2 min. After washing three times with sterile ECM, the embryos were transferred to another sterile beaker with 0.003% bleach solution (0.003% sodium hypochlorite in ECM, filtered using 0.22 μm filter) and incubated at room temperature for 30 min. For germ-free condition, embryos were washed three times with sterile ECM and transferred into 25 cm^2^ flasks with sterile ECM.

At 12 dpf, when all embryos hatched, 200 μL of the culture medium was spread onto the solid culture media to test for bacterial contamination. Larvae were transferred to new 25 cm^2^ flasks with sterile ECM (for germ-free), 5 μm-filtered fish system water (for conventional control), or 1 × 10^6^ CFU/mL *Aeromonas* in sterile ECM (for gnotobiotic) with mashed gamma-irradiated powdered food (Otohime S1, Marubeni Nisshin Feed Co., LTD, Tokyo, Japan).

Two days after culture in respective mediums (14 dpf), 200 μL of the culture medium of all groups was spread onto solid culture media to test for bacterial contamination. The larvae were then transferred to new 25 cm^2^ flasks containing sterile ECM. Subsequently, two days after culturing (16 dpf), the larvae were used for phenotypic analyses.

### 2.11 Bacteria culture from the larval culture medium

A total of 200 μL germ-free, conventional, or gnotobiotic culture medium containing larvae was spread onto solid LB or trypticase soy agar (TSA; Thermo Fisher Scientific) media for aerobic culture at 28°C for 2 days. In addition, anaerobic culture was performed in a solid TSA medium at 28°C for 4 days. AnaeroPack-Anaero (Mitsubishi Gas Chemical, Tokyo, Japan) was used for anaerobic culture, and the anaerobic status was checked using an Anaero-indicator (Mitsubishi Gas Chemical, Tokyo, Japan).

### 2.12 Histological analysis

Whole larvae were fixed in Bouin’s solution overnight. Paraffin sectioning was performed as described in a previous study (31). Coronal sections (5 μm thick) of larval intestines were stained with hematoxylin and eosin. For Periodic acid-Schiff (PAS) staining, the deparaffinized sections were placed in a 0.5% periodic acid solution (Fujifilm Wako, Osaka, Japan) for 5 min and rinsed twice with distilled water. Sections were stained with Schiff’s reagent (Fujifilm Wako) for 5 min, washed with a sulfurous acid solution (Fujifilm Wako) three times, rinsed under running tap water, and counterstained with hematoxylin. For acidic mucin staining, deparaffinized sections were placed in 3% acetic acid for 3 min and stained with Alcian blue solution (pH 2.5; Fujifilm Wako) for 30 min. The sections were rinsed under running tap water and counterstained with hematoxylin. Images were captured using a DP72 camera (Olympus) under a BX60 microscope (Olympus).

### 2.13 RNA sequencing (RNA-seq) and Gene ontology (GO) analysis

RNA- sequencing (RNA-seq) analysis was performed as described in a previous study (32). Whole bodies of germ-free (pools of two fish, n = 3) and conventional (pools of two fish, n = 3) larvae at 14 dpf were kept in RNAprotect reagent (Qiagen, Hilden, Germany) at 4°C. RNA was extracted using QIAzol (Qiagen), followed by DNase treatment and purification using an RNeasy Micro Kit (Qiagen). RNA-seq libraries were prepared using the Kapa Stranded mRNA-Seq Kit (Nippon Genetics, Tokyo, Japan). Six libraries (n = 3/group) were mixed equally and sequenced by Macrogen (Japan) using HiSeqX_Ten (151 bp, paired-end). The obtained raw reads were trimmed using the Trimmomatic software ver. 0.39 with the following parameters: CROP:150, SLIDINGWINDOW:4:15, MINLEN:50 (33). The filtered high-quality reads were mapped to the reference genome using HISAT2 (ver. 2.1.0) (34). The genomic DNA sequence of *Oryzias latipes* from the NCBI database (ASM223467v1) was used as the reference genome. Genes were quantified using StringTie software (ver. 1.3.4). Gene expression variation analysis was performed using Ballgown software (ver. 2.18.0).

GO enrichment analysis was performed for the 53 genes downregulated in germ-free larvae (**Table S3**) using Panther software (version 17.0; http://pantherdb.org/). The analysis was performed using annotation datasets of the complete GO biological processes and the reference list of human genes. Eighteen genes that were uncharacterized or lacked human orthologs were excluded from the analysis.

### 2.14 LPS administration

Ten microliters of LPS (4.5 mg/mL) obtained from *Escherichia coli* O111:B4 (Product Number: L2630, Lot Number: 099M4002V, Sigma-Aldrich, St. Louis, MO) in distilled water was injected into the yolk of 9 dpf (stage 39) embryos. RNA was extracted 2 h post-injection.

## 3 Results

### 3.1 *il2rg* mutation affects lymphocyte development

**Figure 1:**
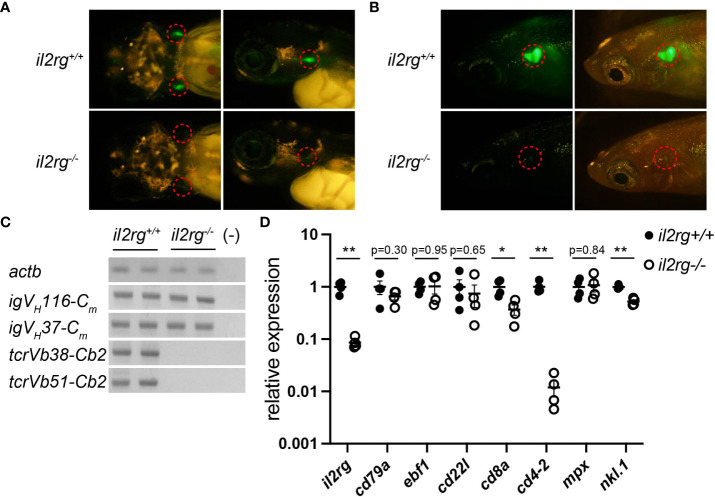
*il2rg* mutation affects T and B cell development. **(A)** *rag1:egfp* expression in larval thymocytes at 10 days post-fertilization (dpf). Dorsal (left) and lateral (right) views of the indicated genotypes. Dotted circles indicate the thymus. **(B)** *rag1:egfp* expression in adult thymocytes at two months post-fertilization (mpf). Lateral views of indicated genotypes with (left) and without (right) visible light transmission. Dotted circles indicate the thymus. Note a few EGFP-positive thymocytes in *il2rg* mutants. **(C)** Reverse transcription PCR (RT-PCR) of whole kidney cells of the indicated genotypes using indicated primers to detect VDJ recombined *igm* and *tcrb*. Two fish for each genotype were used. *actb* expression was used as standard. (−); water control. **(D)** Quantitative PCR (qPCR) analysis of whole kidney cells of the indicated genotypes (n = 4, respectively). The average expression level in *il2rg^+/+^* is normalized as 1. Data represent the mean ± standard error of the mean (SEM). *, p < 0.05; **, p < 0.01 determined using unpaired two-tailed t-test. Results represent one of the two independent experiments with similar results.

To assess the effects of a defective adaptive immune system on the intestinal microbiota of medaka, immunodeficient medaka (*il2rg^-/-^*) were generated by genome editing using the CRISPR/Cas9 system, targeting exon 3 of *il2rg* (**Figure S1A**). A mutant line with a 4 bp deletion in *il2rg* was established, which was predicted to cause a frameshift and encode only 75 appropriate amino acids, followed by 12 unrelated amino acids and a stop codon (**Figure S1B**). The resulting protein lacked most of the fibronectin type III domain (FNIII) and the entire transmembrane domain (TM) (**Figure S1C**) and was considered a null allele. The homozygous form of this frameshift mutant did not exhibit any gross developmental defects. However, crossing the mutant with *rag1:egfp* transgenic medaka, which expresses EGFP under the control of the immature lymphocyte-specific *rag1* promoter (22), revealed a severe reduction in EGFP-expressing cells in the larval thymus, suggesting defects in T cell development (**Figure 1A**). This severe reduction in T-cells was continuously observed in the adult thymus (**Figure 1B**).

Next, we investigated the transcripts of rearranged lymphocyte antigen receptors in the kidney, equivalent to the mammalian bone marrow as a site of hematopoiesis. Reverse transcription PCR **(**RT-PCR) analysis detected VDJ-recombined *igm* transcripts in B cells but not *tcrb* transcripts in the il2rg mutant (**Figure 1C**). qPCR analysis of adult kidney cells revealed a severe reduction in *il2rg* expression in *il2rg* mutants (**Figure 1D**). Under these conditions, the expression of pan B cell marker *cd79*, early B cell marker *ebf1*, and relatively mature B cell marker *cd22l* in the WT and *il2rg* mutant did not differ significantly (**Figure 1D**). In contrast, the expression of T cell markers *cd8a* and *cd4-2* was significantly decreased in *il2rg* mutants **(** **Figure 1D**). Among other blood populations, the expression of the neutrophil marker *mpx* was comparable, but that of the natural cell maker *nkl.1* was significantly reduced in *il2rg* mutants than those in WT medaka (**Figure 1D**). These results suggest that *il2rg* mutation affects T and NK cells, as observed in the zebrafish *il2rg* mutants (11, 27); nevertheless, they grew to adult stage in filtered water with a weekly water change (mortality rate at 3 mpf: 0/15), despite being sensitive to lower water quality (mortality rate: 11/14 in a recirculating system sharing water with other medaka tanks).

### 3.2 Dynamic change of frequency of intestinal microbiota in *il2rg* mutant adult medaka

**Figure 2:**
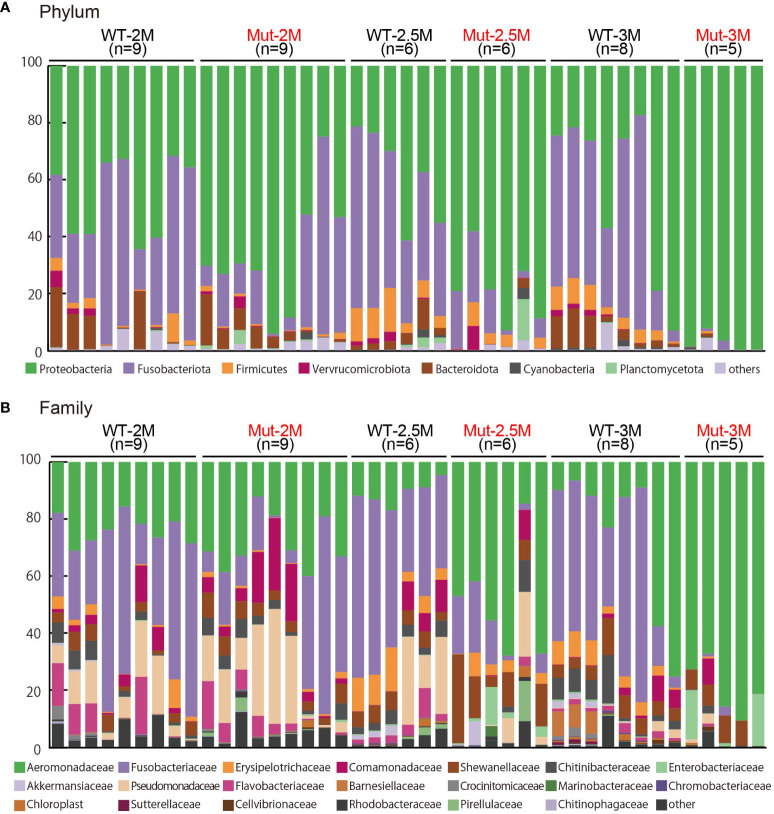
Microbiota analysis of intestines from adult wild type and *il2rg* mutant medaka. **(A, B)** The intestinal bacterial composition of adult wild-type (WT) and *il2rg* mutant (Mut) medaka at 2, 2.5, and 3 months post-fertilization (M). The number of fish in each group (n) is indicated. **(A)** The 100% stacked bar charts indicate the phyla of all bacteria. **(B)** The 100% stacked bar charts indicate the family of all bacteria.

**Figure 3:**
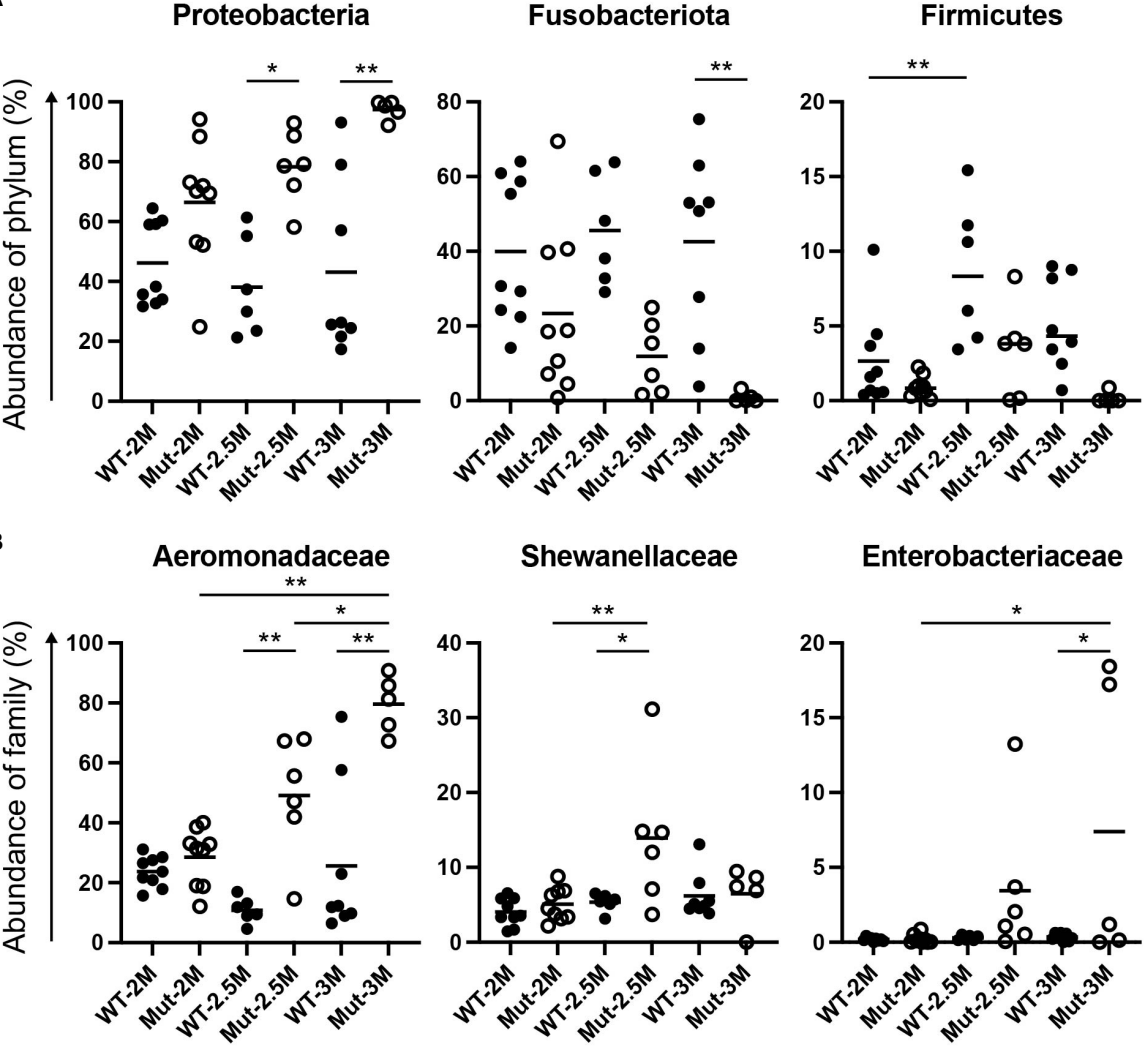
Dynamic change of intestinal bacterial proportions in *il2rg* mutants. **(A, B)** Proportional changes of intestinal bacteria of adult wild-type (WT) and *il2rg* mutant (Mut) medaka at 2, 2.5, and 3 months post-fertilization (M). Relative abundances of **(A)** each phylum and **(B)** each family in all bacteria. Data represent the mean ± SEM. *, p < 0.05; **, p < 0.01 determined using Dunn test with Bonferroni correction. The number of fish in each group is as follows; WT-2M: n = 9, Mut-2M: n = 9, WT-2.5M: n = 6, Mut-2.5M: n = 6, WT-3M: n = 8, Mut-3M: n = 5.

The analysis of the intestinal bacterial composition of the WT and *il2rg* mutants at 2, 2.5, and 3 mpf revealed that most OTUs were represented by Proteobacteria, Fusobacteriota, and Firmicutes at the phylum level in both the WT and *il2rg* mutants at all time points (**Figures 2A**, **3A**). However, the abundance of Proteobacteria was significantly higher in the *il2rg* mutants than that in WT at 2.5 and 3 mpf. Especially at 3 mpf, more than 90% of the intestinal microbiota of *il2rg* mutants comprised Proteobacteria. Furthermore, the abundance of Fusobacteriota was inversely correlated with that of Proteobacteria.

At the family level, Aeromonadaceae was abundant in WT adult medaka intestines (**Figures 2B**, **3B**), which was consistent with the previous report (25). The abundance of Aeromonadaceae gradually increased in the *il2rg* mutants at 2.5 and 3 mpf and was significantly higher than that in the WT (**Figures 2B**, **3B**). This enrichment of Aeromonadaceae was observed repeatedly in independent experiments (**Table S2**). Other significant changes at the family level included a temporary increase in Shewanellaceae at 2.5 mpf and an increase in Enterobacteriaceae at 3 mpf in *il2rg* mutants.

### 3.3 Dynamic change of evenness of intestinal microbiota in *il2rg* mutant adult medaka

**Figure 4:**
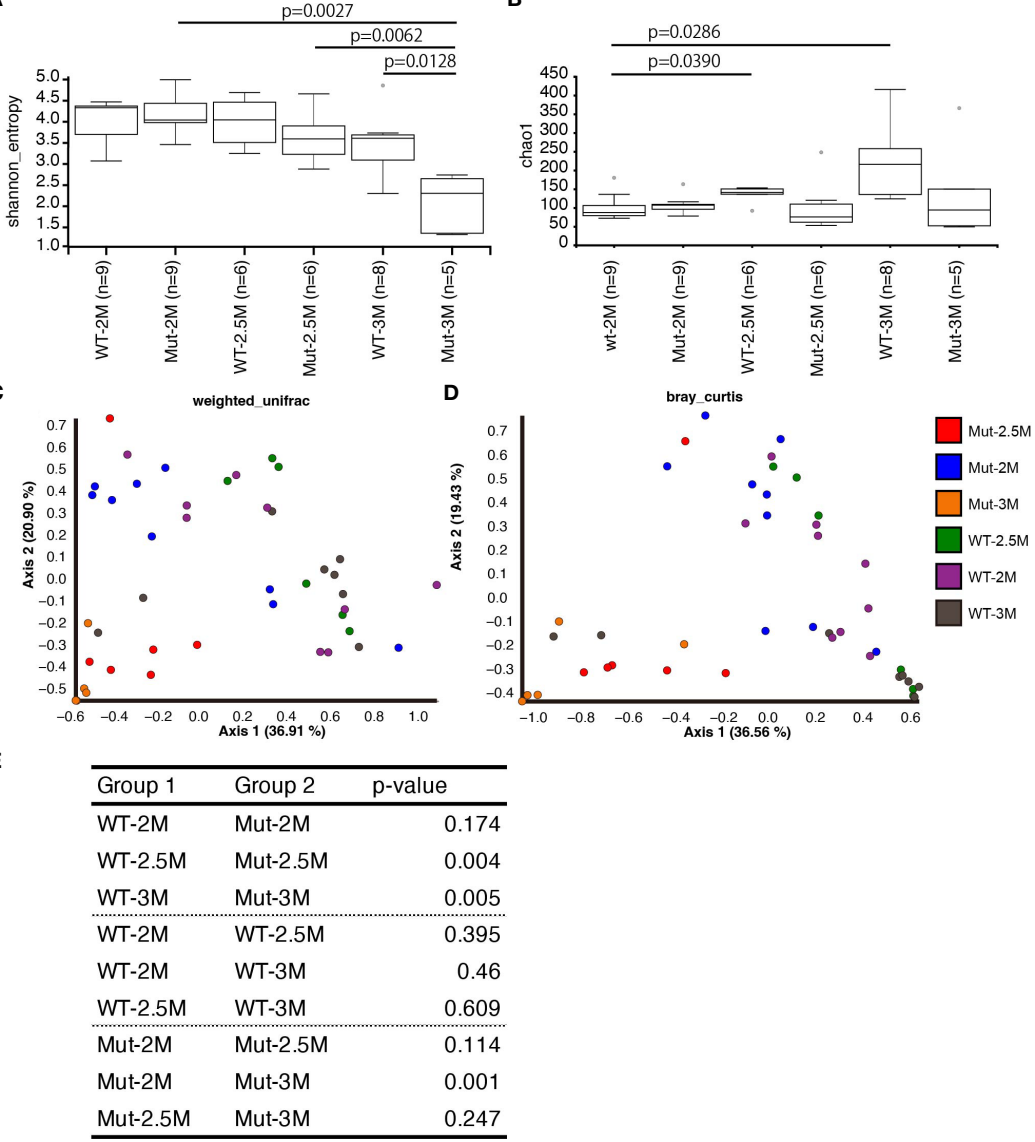
Dynamic change of intestinal bacterial composition in *il2rg* mutant. **(A, B)** Alpha diversity analysis of intestinal bacteria composition of wild-type (WT) and *il2rg* mutant (Mut) medaka at the indicated age (months post-fertilization, M) using Shannon **(A)** and Chao1 indices **(B)**. The number of fish in each group (n) is indicated. **(C, D)** Beta diversity analysis of intestinal bacteria composition of WT and *il2rg* mutants at the indicated age using weighted UniFrac **(C)** and Bray–Curtis plots **(D)**. The number of fish in each group (n) is identical to **(A, B)**. **(E)** The statistical significances of weighted UniFrac **(C)** distances were calculated using PERMANOVA.

To compare the overall composition of intestinal bacteria between the WT and *il2rg* mutants at each time point, alpha diversity indices were analyzed. Shannon indices, which indicate the evenness of OTUs, showed that the WT was unchanged at all three time points (**Figure 4A**). Although the evenness of the OTUs in the *il2rg* mutant was comparable to that of the WT at 2 and 2.5 mpf, it was significantly decreased at 3 mpf (**Figure 4A**), which mainly reflected the increased abundance of Aeromonadaceae. In contrast, Chao1 indices, which indicate the richness of OTUs, were comparable between WT and *il2rg* mutants at all time points, although the index of WT medaka at 3 mpf was higher than that at earlier time points (**Figure 4B**).

Next, we quantified the similarity of bacterial composition among each fish and each group. Analysis of beta-diversity indices using weighted UniFrac and Bray–Curtis plots revealed a stable pattern in WT at all time points. In contrast, the intestinal bacterial composition of *il2rg* mutants showed dynamic shifts at 2.5 and 3 mpf (**Figures 4C, D**). Considering the phylogenetic distances to the datasets, weighted UniFrac indices were chosen for the quantitative measurement of OTUs. The calculation of the distances indicated that the bacterial composition of *il2rg* mutants was significantly distant from that of the WT at 2.5 and 3 mpf (**Figure 4E**).

### 3.4 Effect of *Aeromonas* as a culturable main component of intestinal bacteria on adult medaka

Next, we identified readily culturable bacteria derived from the medaka intestine. A culture of aseptically harvested intestines from adult WT medaka under aerobic conditions revealed uniform shapes and colors of the colonies (**Figure S2A**). The 16S rRNA gene sequences from the 16 colonies were grouped into three types: type A (13 colonies), type B (two colonies), and type C (one colony). The phylogenetic tree of the 16S rRNA gene sequences of the *Aeromonas* family showed that all three types were close to *Aeromonas* and away from *Oceanimonas* (**Figure S2B**). Type A and C sequences were the closest to those of *A. intestinalis*, recently isolated from human feces (35). The type B sequence was identical to the FPC-0866 strain, derived from eels and defined as *A. hydrophila* (36). However, *A. hydrophila* strain ATCC 7966 was relatively phylogenetically distant from the FPC-0866 strain, indicating that the resolution of 16S rRNA gene sequencing was not high enough to identify the species of *Aeromonas* precisely. Nevertheless, all three types are close to *Aeromonas*; therefore, we considered *Aeromonas* a representative bacterium of adult medaka.

To examine the effect of intestine-derived *Aeromonas* on adult medaka, WT medaka were exposed to 6.5 × 10^7^ CFU/mL of one of the type A clones. Although this exposure did not cause lethality (14/14), exposed fish commonly showed vasodilation of the liver and an enlarged spleen (**Figure S2C**), suggesting an inflammatory response. FCM analysis of kidney and spleen cells showed that the exposure did not change the populations of kidney cells; however, the lymphoid population overwhelmed the myeloid population in the spleen of the exposed fish (**Figures S2D, S2E**), implying lymphocyte involvement in the antibacterial response.

### 3.5 Establishment of germ-free condition of medaka

Next, we sought to establish a method for maintaining medaka larvae under germ-free conditions. To this end, we followed one of the protocols for zebrafish germ-free culture (14) with some modifications for medaka. PVP-I and bleach treatment of eggs at 6 dpf before hatching and culture of eggs and larvae thereafter with sterile AB kept the larvae sterile, which was confirmed by the absence of colonies after the larval culture medium was spread on LB agar medium for aerobic culture and TSA agar medium for aerobic and anaerobic cultures (**Figure S3**). Gnotobiotic conditions were also achieved by adding 1 × 10^6^ CFU/mL *Aeromonas* derived from adult medaka intestine to a germ-free culture.

### 3.6 Intestinal epithelial cell development of germ-free medaka

Next, to analyze the structural changes in the intestines of wild-type germ-free larvae, we compared the anterior, middle, and posterior intestinal structures (37) under germ-free, conventional, and gnotobiotic conditions. The anterior intestine contained small absorptive vacuoles in its epithelial layer. However, no apparent structural differences were observed between the conventional, germ-free, and gnotobiotic conditions. No structural differences were observed in the posterior intestines (**Figure S4**).

**Figure 5:**
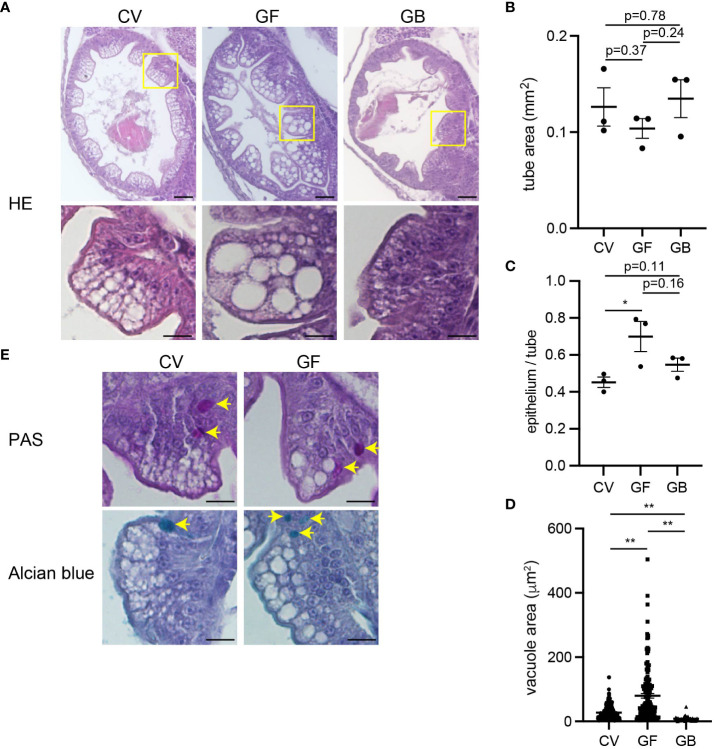
Immature intestinal epithelium in germ-free condition. **(A)** Coronal sections of middle intestines after larval culture in conventional (CV), germ-free (GF), and gnotobiotic (GB) conditions. Enlarged images of the area of yellow squares are shown below. The absorptive vacuoles in the enterocytes in germ-free conditions are enlarged. Scale bars: 50 μm (top) and 20 μm (bottom). **(B)** Comparable middle intestinal tube areas in each larval culture condition. Data indicates the average of three sections per fish; n = 3, respectively. **(C)** Increased thickness of middle intestinal epithelium in germ-free conditions. Ratios of epithelium area vs. total epithelial tube area are shown. Data indicates the average of three sections per fish; n = 3, respectively. **(D)** Increased absorptive vacuole sizes in germ-free conditions. Data represent the mean ± SEM. Statistical significance was determined using an unpaired two-tailed t-test. The number of vacuoles measured is as follows; CV: n = 243, GF: n = 143, GB: n = 44. **(E)** Periodic acid-Schiff (PAS) (top) and Alcian blue (bottom) staining of mid intestines. Arrows indicate stained goblet cells. The absorptive vacuoles are away from goblet cells with PAS-positive neutral mucin and Alcian blue-positive acidic mucin. Scale bar, 20 μm.

However, apparent structural differences were observed in the middle intestine. In the germ-free intestine, although the thickness of the intestinal tube itself was comparable to that in the conventional condition (**Figures 5A, B**), the thickness of the epithelial layers was significantly increased compared to that in the conventional condition (**Figures 5A, C**). Close observation of the germ-free epithelium revealed enlarged absorptive vacuoles. Although such vacuoles were also present in the conventional intestine, their number was significantly higher in the germ-free intestine (**Figures 5A, D**). These vacuole structures were not goblet cells because they did not stain with PAS (for neutral mucin) or with Alcian blue (for acidic mucin) (**Figure 5E**). The increased epithelium thickness and absorptive vacuole enlargement were negated by adding *Aeromonas* bacteria to the gnotobiotic conditions (**Figures 5A, C, D**).

### 3.7 Decreased expression of LPS-dependent defense response genes in germ-free condition

**Figure 6:**
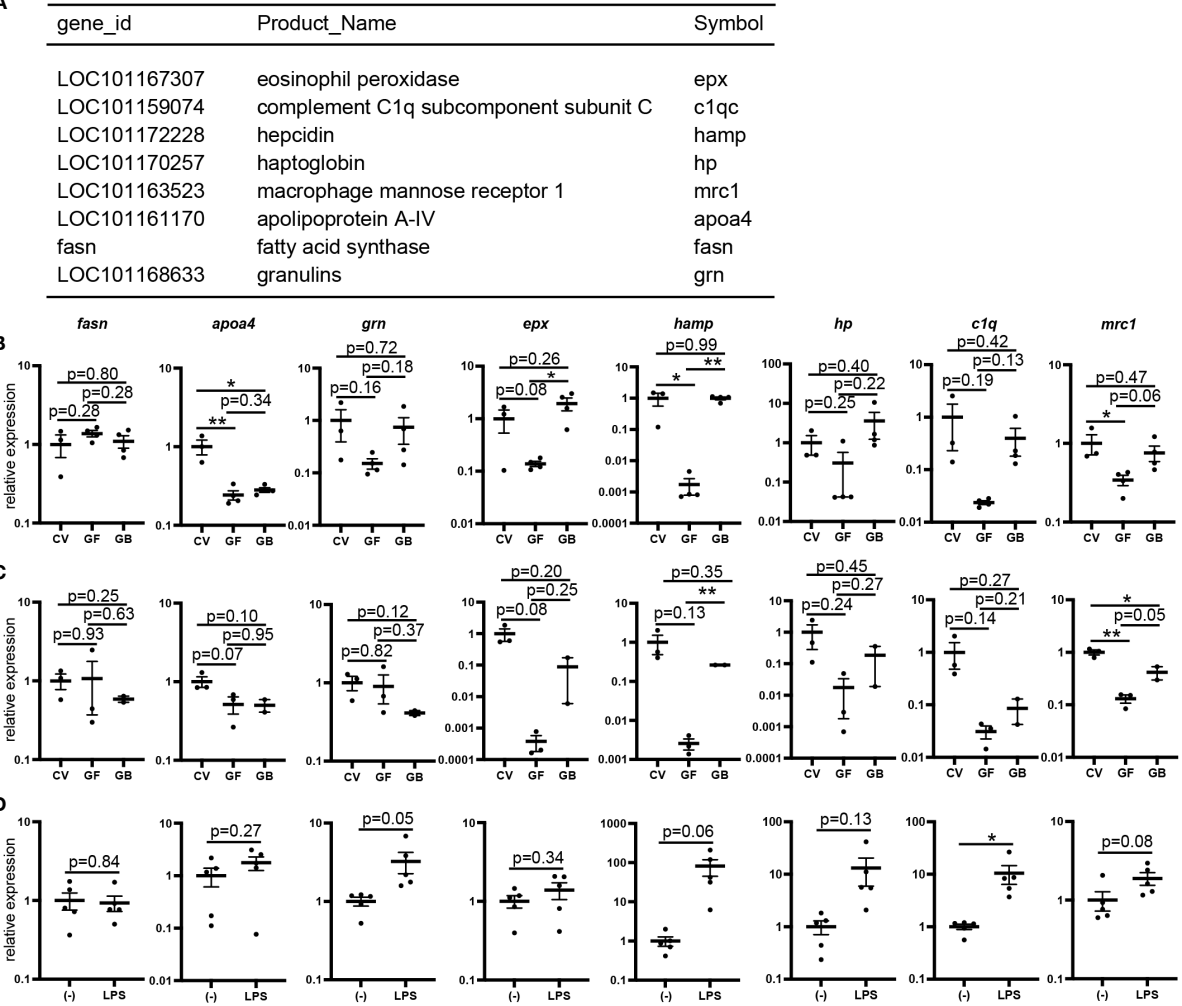
Reduced expression of lipopolysaccharide (LPS)-sensitive genes in germ-free conditions. **(A)** List of defense response (GO:0006952) genes whose expression is decreased in whole bodies of germ-free larvae identified using gene ontology analysis compared to that in conventional larvae (Related data shown in **Tables S3** and **S4**). **(B, C)** Quantitative PCR (qPCR) analysis of defense response genes of 16 days post-fertilization (dpf) larvae in conventional (CV), germ-free (GF), and gnotobiotic (GB) conditions. Whole bodies (CV: n = 3, GF: n = 3, GB: n = 4) **(B)** and intestines (CV: n = 3, GF: n = 3, GB: n = 2) **(C)**. Results represent one of the two independent experiments with similar results. **(D)** qPCR analysis of the defense response genes of whole bodies of LPS- or mock-stimulated (–) embryos at 9 dpf (n = 5, respectively). Results represent one of the three independent experiments with similar results. qPCR data represent the mean ± SEM. *, p < 0.05; **, p < 0.01 determined using unpaired two-tailed t-test.

To investigate the effects of immune-related gene expression in germ-free medaka, RNA-seq analysis of whole bodies of medaka larvae grown under conventional or germ-free conditions was performed. Fifty-three genes were significantly downregulated, and six were significantly upregulated in germ-free larvae (**Tables S3, S4**). Gene ontology analysis of the downregulated genes showed significant enrichment of defense response (GO:0006952) genes (p = 0.0018; **Figure 6A**), which included the regulation of intestinal absorption (GO:1904478) genes (*hamp* and *apoa4a*) (p = 0.00024).

qPCR analysis of whole bodies of germ-free larvae showed a tendency to decrease the expression of seven genes (*grn*, *epx*, *hamp*, *hp*, *c1q*, *mrc1*, and *apoa4*), except *fasn* under germ-free conditions compared to conventional conditions (**Figure 6B**). Interestingly, the expression of *grn*, *epx*, *hamp*, *hp*, *c1q*, and *mrc1*, excluding *apoa4*, was partially recovered under gnotobiotic conditions (**Figure 6B**). qPCR analysis using intestinal RNA samples showed identical expression patterns— the expression of five genes (*epx*, *hamp*, *hp*, *c1q*, and *mrc1*), except *grn* decreased in germ-free conditions and recovered in gnotobiotic conditions (**Figure 6C**). qPCR analysis of whole bodies of larvae injected with LPS, a toll-like receptor 4 (TLR4) signaling inducer, showed increased *hamp*, *hp*, and *c1q* expression (**Figure 6D**), suggesting that the expression of these genes is affected by the detection of bacterial components by TLRs.

## 4 Discussion

In this study, we showed that reciprocal interactions between the microbiome and the intestinal tract are required for the maturation of the medaka immune system using *il2rg* mutant and the establishment of germ-free conditions.

In humans, *il2rg (γc)* is responsible for X-SCID (8). Although receptor complexes utilizing IL2RG and the cytokines serving as these ligands have not been completely identified in teleosts, *il2rg* is expected to be involved in important cytokine signaling pathways for lymphocyte development and function. A recent study in zebrafish showed that *il2rg* mutation affects T and NK cell development (11, 27); concordantly, medaka *il2rg* mutants reported in this study showed a similar phenotype to that of the zebrafish mutant (**Figure 1**). The lack of T cells likely affects B cell activation by T-dependent antigens (38), as observed in mammals, leading to overall defects in adaptive immunity. As *il2rg* is expressed by pan-B cells which are unaffected by *il2rg* mutation, as well as T and NK cells in the kidney (data not shown), a lower expression level of *il2rg* in *il2rg* mutant does not reflect the depletion of *il2rg*-expressing cells, but a reduction of *il2rg* mRNA in each cell, possibly because of nonsense-mediated mRNA decay.

In our chronological analysis of intestinal microbiota, the intestinal bacterial composition in WT medaka was stable at 2–3 mpf (**Figures 2**, **3**), with a predominance of Proteobacteria (46.2 ± 4.7% at 2 mpf), especially Aeromonadaceae family (23.7 ± 1.7%). This result is similar to that of a previous report (25), indicating that the Aeromonadaceae family is the major component of medaka intestinal flora.

To compare the microbiota composition between the WT and *il2rg* mutants, we cohoused both genotypes. As we observed a highly variable gut microbiota composition among the discrete fish tanks (data not shown), cohousing could overcome this problem by focusing on genotype discrepancies. Furthermore, co-housing organisms, including fish, possessing a wide variety of microorganisms, is a common phenomenon in the natural environment.

While changes in the gut microbiota composition have been reported in zebrafish *il2rg* and *rag1* mutants (10, 11), the present study using *il2rg* mutant medaka was carried out in-depth using chronological analysis. Although the gut bacterial composition of the *il2rg* mutant was similar to that of the wild type at 2 mpf, it showed a dynamic change in the following 1 month with a significant predominance of Proteobacteria, mainly Aeromonadaceae (**Figure 3**). This increased abundance of Aeromonadaceae led to reduced evenness of OTUs in the mutants, even though richness itself was unchanged (**Figure 4**). Hence, in contrast to earlier time points, the intestinal bacterial composition pattern in the *il2rg* mutant was dramatically and significantly changed at 3 mpf, suggesting that a deficiency in T and NK cells affects the intestinal bacterial composition. Although reduced bacterial diversity has also been observed in the zebrafish *il2rg* mutant (11), the abundance of Aeromonadaceae showed an opposite trend in these two species—zebrafish showed an increased abundance of Firmicutes. This contrast could be because of the physiological differences between them.

We cloned *Aeromonas* as the main intestinal bacteria (**Figure S2**). *Aeromonas* is a genus comprising several gram-negative rod-shaped bacteria, including *A. hydrophila* and *A. veronii* and is present in the gut as opportunistic bacteria (39, 40). Exposure to the *A. hydrophila* FPC-0866 strain derived from the Japanese eel was reported to cause lethality in an apoptosis-associated speck-like (ASC) protein (a component of the inflammasome) coding gene mutant (36). Phylogenetic analysis of the medaka intestine-derived *Aeromonas* clones based on 16S rRNA gene sequences indicated a high similarity to FPC-0866 (**Figure S2**). The inflammatory phenotype of the liver and spleen and an increase in the lymphoid population in the spleen after exposure to type A clones of *Aeromonas* suggest the harmful effects of this bacterium at high concentrations. It is possible that the overwhelming *Aeromonas* in immunodeficient *il2rg* mutants is one of the reasons for lethality in early adulthood.

Some fish species have been reported to lack the principal components of adaptive immunity; Atlantic cod does not have CD4^+^ T cells or their related genes (41) and some anglerfish species engage in permanent sexual parasitism and even lack *rag*-based adaptive immunity (42). In particular, anglerfish are likely to control environmental microorganisms primarily through innate immunity. In addition, innate immunity is thought to be more important than adaptive immunity in fish (43, 44) and has been shown to control intestinal microbiota composition in zebrafish (9). However, the present study, together with other studies in zebrafish (10, 11) showed that the maintenance of healthy gut microbiota also depends on adaptive immunity.

T cell development in teleosts occurs in waves. For zebrafish, T cells colonize the intestine as early as 5dpf and increase during inflammation (45); a second wave begins to colonize the larva at around 21 dpf (46, 47). In zebrafish *rag1* mutants, a different intestinal microbial community has been reported compared to its WT siblings at 14 wpf, represented by enrichment of *Vibrio* (10). Although microbiota analysis was not done at earlier timepoints, qPCR analysis indicated that enrichment of *Vibrio* was apparent at 5 wpf. Together, these observations suggest that T cells colonizing the periphery during the first and subsequent waves may have little immediate impact on the composition of the intestinal microbiota. Whether this is because re-organization of the microbiota is a slow process or because subtle changes may escape detection remains to be clarified. In any case, our *il2rg* mutant medaka showed a change in microbial composition only after 2.5 mpf (**Figures 2** **–** **4**). Although evidence for T cell colonization of the larval intestine in medaka has been provided (48), it is possible that the microbiota also changes during waves of T cell development. These findings suggest that the detectable change of composition of the microbiota in fish lacking T and NK cells only at 2.5 mpf could be related to the developmental regulation of these cell lineages. The cellular mechanisms for microbiota regulation, including the timing of second-wave T cell development in medaka, are topics for future work.

We established the method for growing medaka larvae in germ-free and gnotobiotic conditions for the first time. Under germ-free conditions, the absorptive vacuole intestinal epithelium in the mid-intestine of the larvae was significantly larger than that of those under the conventional and gnotobiotic conditions (**Figure 5**). Large absorptive vacuoles are observed in the immature and occasionally pathogenic epithelium in mammals (49, 50), suggesting that intestinal epithelium maturation is blocked or delayed in germ-free larvae. As germ-free culture was carried out up to 15 dpf in this study, the phenotypic changes in the intestinal epithelium after longer germ-free cultures were unclear. However, this is feasible for future studies.

RNA-Seq analysis showed that the genes downregulated under germ-free conditions were enriched in those involved in defense responses (**Figure 6**). Three out of eight genes, *hamp*, *hp*, and *c1q*, were downregulated in germ-free conditions; however, their expression was recovered in gnotobiotic conditions in both whole bodies and intestines and was dependent on *in vivo* LPS stimulation. Consistently, in mammals, these three genes are upregulated by LPS stimulation via TLR4 (51–55). In particular, *hamp* expression was more than 300 times downregulated in germ-free whole bodies and intestines. In addition, LPS stimulation induced *hamp* expression more than 80 times. These findings are consistent with a previous study in mouse myeloid cells, which showed that *hamp* is induced by LPS through TLR4 (51). The antimicrobial peptide hepcidin, encoded by *hamp*, has antiviral activity in medaka (56). In addition, *in vitro* antiviral, antibacterial, and antitumor activities of marine medaka (*O. melastigmus*) have been reported (57), suggesting the importance of this antimicrobial peptide upon exposure of medaka larvae to microorganisms. The main function of *hp* is to bind to hemoglobin released from damaged erythrocytes; nevertheless, *hp* also promotes immune tolerance to LPS by suppressing proinflammatory cytokines (52, 53). *c1q*, a component of the complement C1 complex, suppresses LPS-induced pro-inflammatory cytokine production in dendritic cells and monocytes in mice (54, 55). These results suggest that the reduction in the expression of these genes under germ-free conditions was due to a lack of bacteria-induced TLR signaling. In addition, the downregulated expression of *mrc1*, a marker of M2 macrophages activated by IL-4 (58) and independent of M1 macrophages through LPS stimulation, was also restored under gnotobiotic conditions, which likely reflects that *mrc1* expression was only mildly upregulated (1.9x) by LPS injection.

The germ-free and gnotobiotic conditions of medaka will be useful for further characterization of the effects of intestinal bacteria on larval physiology. Compared to zebrafish, which hatch before the completion of organogenesis, medaka organogenesis is completed during the embryonic stages. Accordingly, the physiological effects of bacterial exposure may differ among species.

## Data availability statement

The datasets presented in this study can be found in online repositories. The names of the repository/repositories and accession number(s) can be found below: https://www.ddbj.nig.ac.jp/index-e.html, DRA016703 (metagenome analysis) and DRA016696 (RNA-seq analysis).

## Ethics statement

The animal study was approved by Utsunomiya University Animal Experimentation Committee. The study was conducted in accordance with the local legislation and institutional requirements.

## Author contributions

NI: Conceptualization, Data curation, Formal analysis, Funding Acquisition, Investigation, Methodology, Writing-original draft, Writing-review & editing. HS: Data curation, Formal analysis, Investigation, Methodology, Software, Writing-original draft, Writing-review & editing. YS: Data curation, Formal analysis, Investigation, Writing-review & editing. RM: Data curation, Formal analysis, Investigation, Writing-review & editing. JG: Data curation, Formal analysis, Investigation, Writing-review & editing. NY: Data curation, Formal analysis, Investigation, Writing-review & editing. TS: Software, Writing-review & editing. MM: Supervision, Writing-review & editing.

## References

[1] Bäckhed F Ding H Wang T Hooper LV Koh GY Nagy A. The gut microbiota as an environmental factor that regulates fat storage. Proc Natl Acad Sci USA (2004) 101(44):15718–23. doi: 10.1073/pnas.0407076101 PMC524219 15505215

[2] Vijay-Kumar M Aitken JD Carvalho FA Cullender TC Mwangi S Srinivasan S. Metabolic syndrome and altered gut microbiota in mice lacking Toll-like receptor 5. Science (2010) 328(5975):228–31. doi: 10.1126/science.1179721 PMC4714868 20203013

[3] Honda K Littman DR. The microbiota in adaptive immune homeostasis and disease. Nature (2016) 535(7610):75–84. doi: 10.1038/nature18848 27383982

[4] Wen L Ley RE Volchkov PY Stranges PB Avanesyan L Stonebraker AC. Innate immunity and intestinal microbiota in the development of Type 1 diabetes. Nature (2008) 455(7216):1109–13. doi: 10.1038/nature07336 PMC2574766 18806780

[5] Dzutsev A Badger JH Perez-Chanona E Roy S Salcedo R Smith CK. Microbes and cancer. Annu Rev Immunol (2017) 35:199–228. doi: 10.1146/annurev-immunol-051116-052133 28142322

[6] Berbers RM Franken IA Leavis HL. Immunoglobulin A and microbiota in primary immunodeficiency diseases. Curr Opin Allergy Clin Immunol (2019) 19(6):563–70. doi: 10.1097/aci.0000000000000581 31389816

[7] Gereige JD Maglione PJ. Current understanding and recent developments in common variable immunodeficiency associated autoimmunity. Front Immunol (2019) 10:2753. doi: 10.3389/fimmu.2019.02753 31921101 PMC6914703

[8] Noguchi M Yi H Rosenblatt HM Filipovich AH Adelstein S Modi WS. Interleukin-2 receptor gamma chain mutation results in X-linked severe combined immunodeficiency in humans. Cell (1993) 73(1):147–57. doi: 10.1016/0092-8674(93)90167-o 8462096

[9] Burns AR Miller E Agarwal M Rolig AS Milligan-Myhre K Seredick S. Interhost dispersal alters microbiome assembly and can overwhelm host innate immunity in an experimental zebrafish model. Proc Natl Acad Sci USA (2017) 114(42):11181–6. doi: 10.1073/pnas.1702511114 PMC5651736 28973938

[10] Brugman S Schneeberger K Witte M Klein MR van den Bogert B Boekhorst J. T lymphocytes control microbial composition by regulating the abundance of Vibrio in the zebrafish gut. Gut Microbes (2014) 5(6):737–47. doi: 10.4161/19490976.2014.972228 PMC4615293 25536157

[11] Sertori R Jones R Basheer F Rivera L Dawson S Loke S. Generation and characterization of a zebrafish IL-2Rγc SCID model. Int J Mol Sci (2022) 23(4):2385. doi: 10.3390/ijms23042385 35216498 PMC8875600

[12] Murdoch CC Rawls JF. Commensal microbiota regulate vertebrate innate immunity-insights from the zebrafish. Front Immunol (2019) 10:2100. doi: 10.3389/fimmu.2019.02100 31555292 PMC6742977

[13] Pham LN Kanther M Semova I Rawls JF. Methods for generating and colonizing gnotobiotic zebrafish. Nat Protoc (2008) 3(12):1862–75. doi: 10.1038/nprot.2008.186 PMC2596932 19008873

[14] Melancon E Gomez de la Torre Canny S Sichel S Kelly M Wiles TJ Rawls JF. Best practices for germ-free derivation and gnotobiotic zebrafish husbandry. Methods Cell Biol (2017) 138:61–100. doi: 10.1016/bs.mcb.2016.11.005 28129860 PMC5568843

[15] Galindo-Villegas J García-Moreno D de Oliveira S Meseguer J Mulero V. Regulation of immunity and disease resistance by commensal microbes and chromatin modifications during zebrafish development. Proc Natl Acad Sci USA (2012) 109(39):E2605–14. doi: 10.1073/pnas.1209920109 PMC3465450 22949679

[16] Bates JM Akerlund J Mittge E Guillemin K. Intestinal alkaline phosphatase detoxifies lipopolysaccharide and prevents inflammation in zebrafish in response to the gut microbiota. Cell Host Microbe (2007) 2(6):371–82. doi: 10.1016/j.chom.2007.10.010 PMC2730374 18078689

[17] Galindo-Villegas J Montalban-Arques A Liarte S de Oliveira S Pardo-Pastor C Rubio-Moscardo F. TRPV4-mediated detection of hyposmotic stress by skin keratinocytes activates developmental immunity. J Immunol (2016) 196(2):738–49. doi: 10.4049/jimmunol.1501729 26673139

[18] Cheesman SE Neal JT Mittge E Seredick BM Guillemin K. Epithelial cell proliferation in the developing zebrafish intestine is regulated by the Wnt pathway and microbial signaling via Myd88. Proc Natl Acad Sci USA (2011) 108 Suppl 1(Suppl 1):4570–7. doi: 10.1073/pnas.1000072107 PMC3063593 20921418

[19] Koch BEV Yang S Lamers G Stougaard J Spaink HP. Intestinal microbiome adjusts the innate immune setpoint during colonization through negative regulation of MyD88. Nat Commun (2018) 9(1):4099. doi: 10.1038/s41467-018-06658-4 30291253 PMC6173721

[20] Wittbrodt J Shima A Schartl M. Medaka–a model organism from the far East. Nat Rev Genet (2002) 3(1):53–64. doi: 10.1038/nrg704 11823791

[21] Iwanami N Takahama Y Kunimatsu S Li J Takei R Ishikura Y. Mutations affecting thymus organogenesis in Medaka, Oryzias latipes. Mech Dev (2004) 121(7-8):779–89. doi: 10.1016/j.mod.2004.03.020 15210185

[22] Li J Iwanami N Hoa VQ Furutani-Seiki M Takahama Y. Noninvasive intravital imaging of thymocyte dynamics in medaka. J Immunol (2007) 179(3):1605–15. doi: 10.4049/jimmunol.179.3.1605 17641027

[23] Iwanami N. Zebrafish as a model for understanding the evolution of the vertebrate immune system and human primary immunodeficiency. Exp Hematol (2014) 42(8):697–706. doi: 10.1016/j.exphem.2014.05.001 24824573

[24] Bajoghli B. Evolution and function of chemokine receptors in the immune system of lower vertebrates. Eur J Immunol (2013) 43(7):1686–92. doi: 10.1002/eji.201343557 23719857

[25] Okamura Y Morimoto N Ikeda D Mizusawa N Watabe S Miyanishi H. Interleukin-17A/F1 Deficiency Reduces Antimicrobial Gene Expression and Contributes to Microbiome Alterations in Intestines of Japanese medaka (Oryzias latipes). Front Immunol (2020) 11:425. doi: 10.3389/fimmu.2020.00425 32256492 PMC7092794

[26] Okamura Y Kinoshita M Kono T Sakai M Hikima JI. Deficiency of interleukin-17 receptor A1 induces microbiota disruption in the intestine of Japanese medaka, Oryzias latipes. Comp Biochem Physiol Part D Genomics Proteomics (2021) 40:100885. doi: 10.1016/j.cbd.2021.100885 34339936

[27] Sertori R Liongue C Basheer F Lewis KL Rasighaemi P de Coninck D. Conserved IL-2Rγc signaling mediates lymphopoiesis in zebrafish. J Immunol (2016) 196(1):135–43. doi: 10.4049/jimmunol.1403060 26590317

[28] Iwamatsu T. Stages of normal development in the medaka Oryzias latipes. Mech Dev (2004) 121(7-8):605–18. doi: 10.1016/j.mod.2004.03.012 15210170

[29] Wong S Stephens WZ Burns AR Stagaman K David LA Bohannan BJ. Ontogenetic differences in dietary fat influence microbiota assembly in the zebrafish gut. mBio (2015) 6(5):e00687–15. doi: 10.1128/mBio.00687-15 PMC4611033 26419876

[30] Kobayashi I Kondo M Yamamori S Kobayashi-Sun J Taniguchi M Kanemaru K. Enrichment of hematopoietic stem/progenitor cells in the zebrafish kidney. Sci Rep (2019) 9(1):14205. doi: 10.1038/s41598-019-50672-5 31578390 PMC6775131

[31] Imai T Saino K Matsuda M. Mutation of Gonadal soma-derived factor induces medaka XY gonads to undergo ovarian development. Biochem Biophys Res Commun (2015) 467(1):109–14. doi: 10.1016/j.bbrc.2015.09.112 26408909

[32] Iwanami N Ozaki Y Sakaguchi H Watanabe Y Meng Q Matsumoto K. Evolutionarily conserved role of hps1 in melanin production and blood coagulation in medaka fish. G3 (Bethesda) (2022) 12(10):jkac204. doi: 10.1093/g3journal/jkac204 35944207 PMC9526055

[33] Bolger AM Lohse M Usadel B. Trimmomatic: a flexible trimmer for Illumina sequence data. Bioinformatics (2014) 30(15):2114–20. doi: 10.1093/bioinformatics/btu170 PMC4103590 24695404

[34] Pertea M Kim D Pertea GM Leek JT Salzberg SL. Transcript-level expression analysis of RNA-seq experiments with HISAT, StringTie and Ballgown. Nat Protoc (2016) 11(9):1650–67. doi: 10.1038/nprot.2016.095 PMC5032908 27560171

[35] Figueras MJ Latif-Eugenín F Ballester F Pujol I Tena D Berg K. ‘Aeromonas intestinalis’ and ‘Aeromonas enterica’ isolated from human faeces, ‘Aeromonas crassostreae’ from oyster and ‘Aeromonas aquatilis’ isolated from lake water represent novel species. New Microbes New Infect (2017) 15:74–6. doi: 10.1016/j.nmni.2016.11.019 PMC5192473 28050251

[36] Morimoto N Okamura Y Maekawa S Wang HC Aoki T Kono T. ASC-deficiency impairs host defense against Aeromonas hydrophila infection in Japanese medaka, Oryzias latipes. Fish Shellfish Immunol (2020) 105:427–37. doi: 10.1016/j.fsi.2020.07.027 32712229

[37] Hedrera MI Galdames JA Jimenez-Reyes MF Reyes AE Avendaño-Herrera R Romero J. Soybean meal induces intestinal inflammation in zebrafish larvae. PloS One (2013) 8(7):e69983. doi: 10.1371/journal.pone.0069983 23894568 PMC3720926

[38] Mond JJ Vos Q Lees A Snapper CM. T cell independent antigens. Curr Opin Immunol (1995) 7(3):349–54. doi: 10.1016/0952-7915(95)80109-x 7546399

[39] Janda JM Abbott SL. The genus Aeromonas: taxonomy, pathogenicity, and infection. Clin Microbiol Rev (2010) 23(1):35–73. doi: 10.1128/cmr.00039-09 20065325 PMC2806660

[40] Chandrarathna H Nikapitiya C Dananjaya SHS Wijerathne CUB Wimalasena S Kwun HJ. Outcome of co-infection with opportunistic and multidrug resistant Aeromonas hydrophila and A. veronii in zebrafish: Identification, characterization, pathogenicity and immune responses. Fish Shellfish Immunol (2018) 80:573–81. doi: 10.1016/j.fsi.2018.06.049 29964197

[41] Star B Nederbragt AJ Jentoft S Grimholt U Malmstrøm M Gregers TF. The genome sequence of Atlantic cod reveals a unique immune system. Nature (2011) 477(7363):207–10. doi: 10.1038/nature10342 PMC3537168 21832995

[42] Swann JB Holland SJ Petersen M Pietsch TW Boehm T. The immunogenetics of sexual parasitism. Science (2020) 369(6511):1608–15. doi: 10.1126/science.aaz9445 32732279

[43] Wienholds E Schulte-Merker S Walderich B Plasterk RH. Target-selected inactivation of the zebrafish rag1 gene. Science (2002) 297(5578):99–102. doi: 10.1126/science.1071762 12098699

[44] Tokunaga Y Shirouzu M Sugahara R Yoshiura Y Kiryu I Ototake M. Comprehensive validation of T- and B-cell deficiency in rag1-null zebrafish: Implication for the robust innate defense mechanisms of teleosts. Sci Rep (2017) 7(1):7536. doi: 10.1038/s41598-017-08000-2 28790360 PMC5548773

[45] Coronado M Solis CJ Hernandez PP Feijóo CG. Soybean meal-induced intestinal inflammation in zebrafish is T cell-dependent and has a th17 cytokine profile. Front Immunol (2019) 10:610. doi: 10.3389/fimmu.2019.00610 31001250 PMC6454071

[46] Schorpp M Bialecki M Diekhoff D Walderich B Odenthal J Maischein HM. Conserved functions of Ikaros in vertebrate lymphocyte development: genetic evidence for distinct larval and adult phases of T cell development and two lineages of B cells in zebrafish. J Immunol (2006) 177(4):2463–76. doi: 10.4049/jimmunol.177.4.2463 16888008

[47] Hess I Sagar C Grün D Schorpp M Boehm T. Stage-specific and cell type-specific requirements of ikzf1 during haematopoietic differentiation in zebrafish. Sci Rep (2022) 12(1):21401. doi: 10.1038/s41598-022-25978-6 36496511 PMC9741631

[48] Aghaallaei N Agarwal R Benjaminsen J Lust K Bajoghli B Wittbrodt J. Antigen-presenting cells and T cells interact in a specific area of the intestinal mucosa defined by the ccl25-ccr9 axis in medaka. Front Immunol (2022) 13:812899. doi: 10.3389/fimmu.2022.812899 35185906 PMC8853713

[49] Sureda EA Gidlund C Weström B Prykhodko O. Induction of precocious intestinal maturation in T-cell deficient athymic neonatal rats. World J Gastroenterol (2017) 23(42):7531–40. doi: 10.3748/wjg.v23.i42.7531 PMC5698246 29204053

[50] Remis NN Wiwatpanit T Castiglioni AJ Flores EN Cantú JA García-Añoveros J. Mucolipin co-deficiency causes accelerated endolysosomal vacuolation of enterocytes and failure-to-thrive from birth to weaning. PloS Genet (2014) 10(12):e1004833. doi: 10.1371/journal.pgen.1004833 25521295 PMC4270466

[51] Peyssonnaux C Zinkernagel AS Datta V Lauth X Johnson RS Nizet V. TLR4-dependent hepcidin expression by myeloid cells in response to bacterial pathogens. Blood (2006) 107(9):3727–32. doi: 10.1182/blood-2005-06-2259 PMC1895778 16391018

[52] Raju SM Kumar AP Yadav AN Rajkumar K Mvs S Burgula S. Haptoglobin improves acute phase response and endotoxin tolerance in response to bacterial LPS. Immunol Lett (2019) 207:17–27. doi: 10.1016/j.imlet.2019.01.002 30625342

[53] Krzyszczyk P Kang HJ Kumar S Meng Y O’Reggio MD Patel K. Anti-inflammatory effects of haptoglobin on LPS-stimulated macrophages: Role of HMGB1 signaling and implications in chronic wound healing. Wound Repair Regener (2020) 28(4):493–505. doi: 10.1111/wrr.12814 PMC10927319 32428978

[54] Yamada M Oritani K Kaisho T Ishikawa J Yoshida H Takahashi I. Complement C1q regulates LPS-induced cytokine production in bone marrow-derived dendritic cells. Eur J Immunol (2004) 34(1):221–30. doi: 10.1002/eji.200324026 14971048

[55] Fraser DA Bohlson SS Jasinskiene N Rawal N Palmarini G Ruiz S. C1q and MBL, components of the innate immune system, influence monocyte cytokine expression. J Leukoc Biol (2006) 80(1):107–16. doi: 10.1189/jlb.1105683 16617157

[56] Wang YD Kung CW Chen JY. Antiviral activity by fish antimicrobial peptides of epinecidin-1 and hepcidin 1-5 against nervous necrosis virus in medaka. Peptides (2010) 31(6):1026–33. doi: 10.1016/j.peptides.2010.02.025 20214942

[57] Cai L Cai JJ Liu HP Fan DQ Peng H Wang KJ. Recombinant medaka (Oryzias melastigmus) pro-hepcidin: Multifunctional characterization. Comp Biochem Physiol B Biochem Mol Biol (2012) 161(2):140–7. doi: 10.1016/j.cbpb.2011.10.006 22051539

[58] Orecchioni M Ghosheh Y Pramod AB Ley K. Macrophage Polarization: Different Gene Signatures in M1(LPS+) vs. Classically and M2(LPS-) vs. Alternatively Activated Macrophages. Front Immunol (2019) 10:1084. doi: 10.3389/fimmu.2019.01084 31178859 PMC6543837

