# Supplementary material for: Maturation of the medaka immune system depends on reciprocal interactions between the microbiota and the intestinal tract
Source: Front Immunol. 2023 Sep 12;14:1259519. doi: 10.3389/fimmu.2023.1259519 (PMC10520778; doi:10.3389/fimmu.2023.1259519)
Supplement: Supplementary file 1 [file DataSheet_1.pdf]

Supplementary Material

1.1 Supplementary Figures

Figure S1

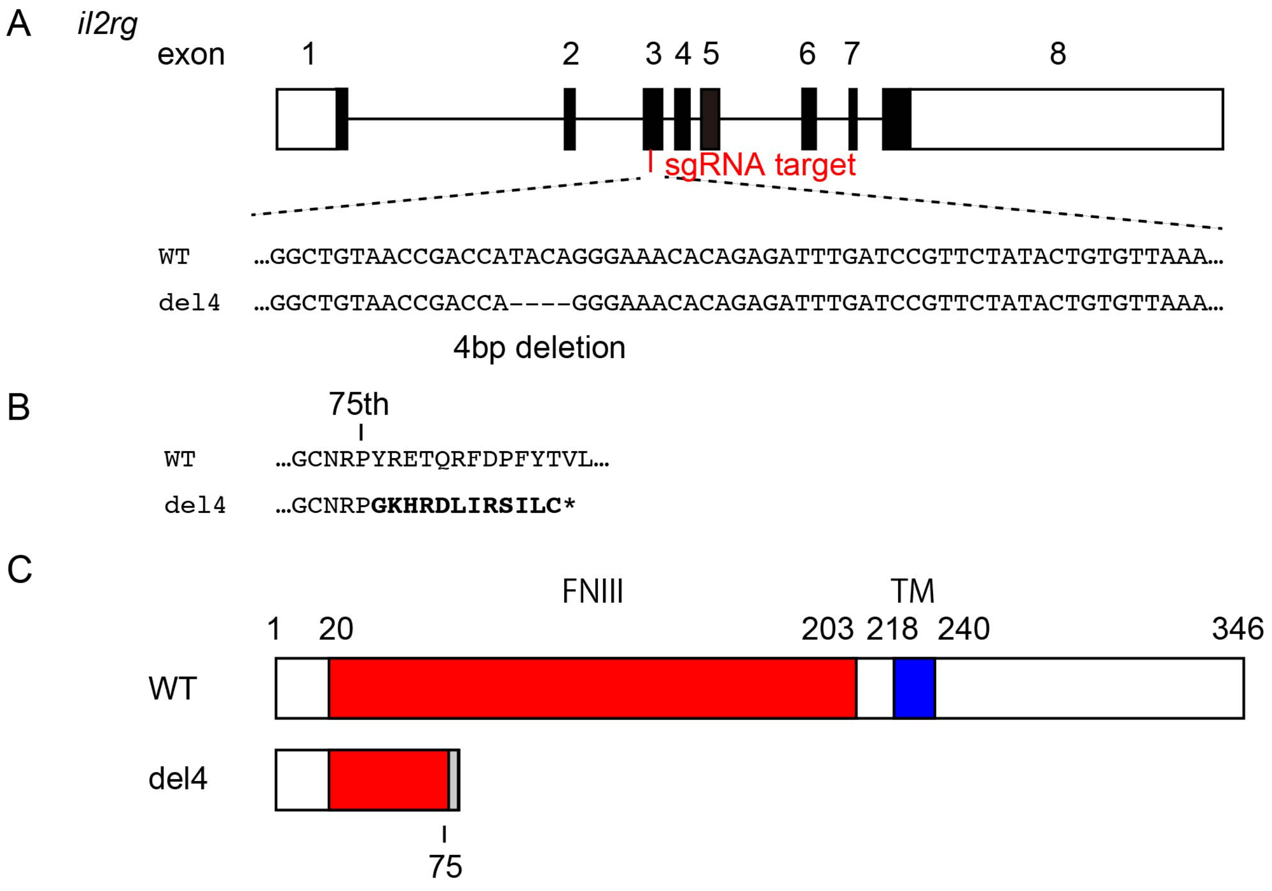

Figure S1

Generation of *il2rg* mutations by genome editing. (A) Exon/intron structure of the medaka *il2rg* gene. Closed boxes indicate coding exons. Open boxes indicate untranslated exons. A sgRNA target for genome editing is indicated in red. 4 bp deletion in exon 3 in mutant *il2rg* is shown at the bottom. (B) Deduced amino acid sequences of wild-type and Il2rg mutant proteins. Bold letters indicate unrelated amino acids. * indicates the stop codon. (C) Domain structures of the wild type and del4 mutant Il2rg proteins. Fibronectin type III domain (FNIII) and transmembrane domain (TM) are indicated in red and blue, respectively.

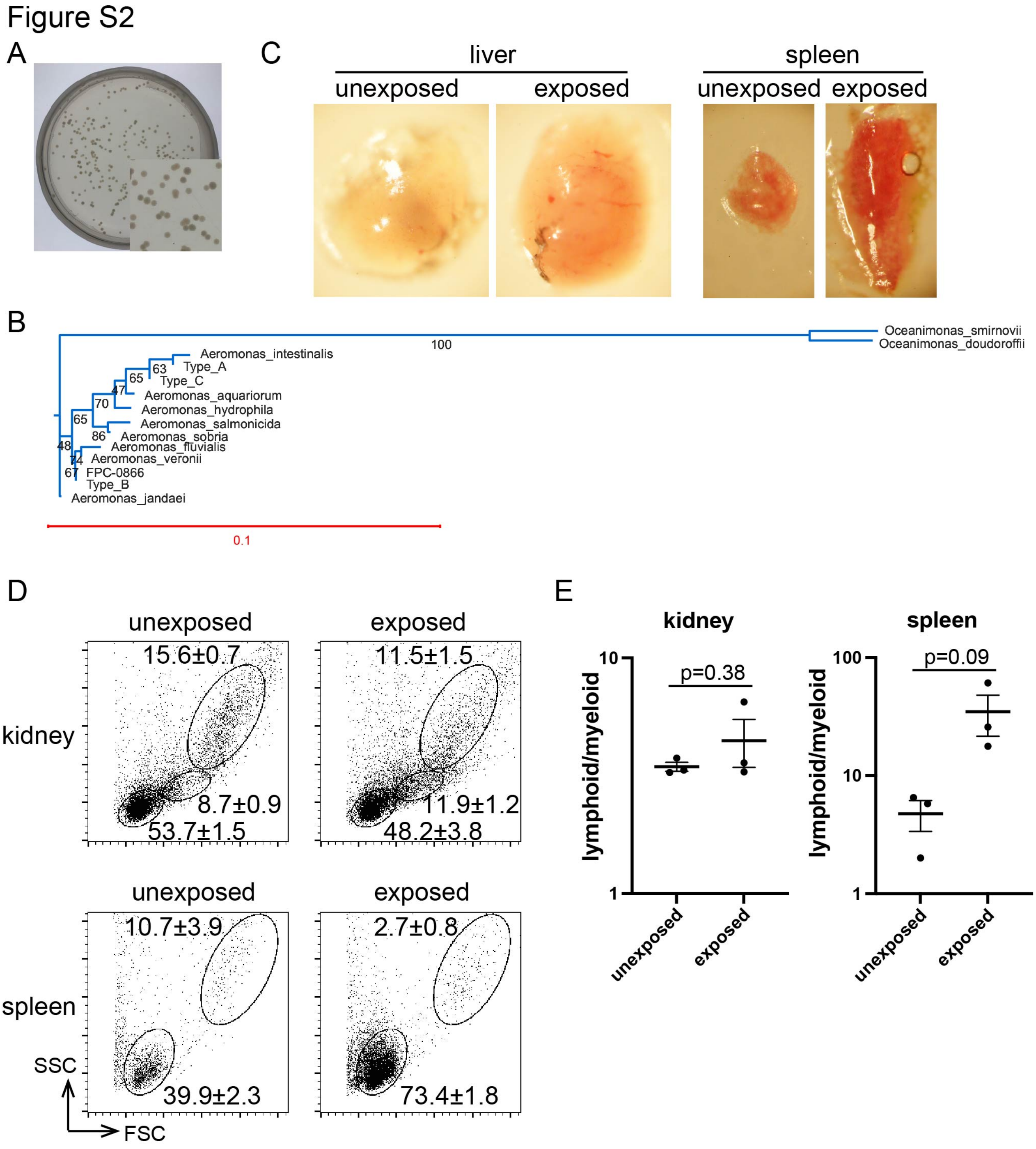

Figure S2

Culturable *Aeromonas* bacteria derived from adult medaka intestine. (A) Colonies on LB agar plate after culture of bacteria isolated from adult medaka intestine in aerobic condition. (B) Phylogenic tree of cultured bacteria derived from wild-type adult intestine. 16S rRNA gene sequences of 16 colonies were grouped into 3 types; Type A (13 colonies), Type B (2 colonies), and Type C (1 colony). FPC-0866 is derived from the Japanese eel (*Anguilla japonica*) and is defined as *Aeromonas hydrophila* [33]. The other components of the tree and their NCBI accession numbers are as follows: *Oceanimonas smirnovii* (AY538714.1), *Oceanimonas_doudoroffii* (AB019390.1), *Aeromonas sobria*, strain NCIMB 12065 (X60412.2), *Aeromonas hydrophila*, strain ATCC 7966 (X60404.2), *Aeromonas aquariorum* strain MDC47 (EU085557.2), *Aeromonas intestinalis*, strain 1178C (LT630759.1), *Aeromonas salmonicida*,

strain NCIMB 1102 (X60405.2), *Aeromonas fluvialis* strain 717 (FJ230078.2), *Aeromonas veronii*, strain ATCC 35624 (X60414.2), *Aeromonas jandaei*, strain ATCC 49568 (X60413.2). Bootstrap values (1000 resampling) are shown at the nodes. The scale bar represents 0.1 substitutions per nucleotide position. (C) Liver and spleen of wild-type medaka. Unexposed and exposed to a clone of intestine-derived *Aeromonas*. Note vasodilation of liver and enlarged spleen in the exposed fish. (D) Flow cytometric profiles of kidney and spleen cells. Unexposed and exposed to a clone of intestine-derived *Aeromonas*. The circles indicate lymphoid, precursor, and myeloid populations. The numbers indicate the mean ± SEM of the proportion (n = 3, respectively). (E) Plots of lymphoid/myeloid ratio of kidney and spleen (n = 3, respectively). Data represent the mean ± SEM; statistical significance was determined using an unpaired two-tailed t-test.

Figure S3

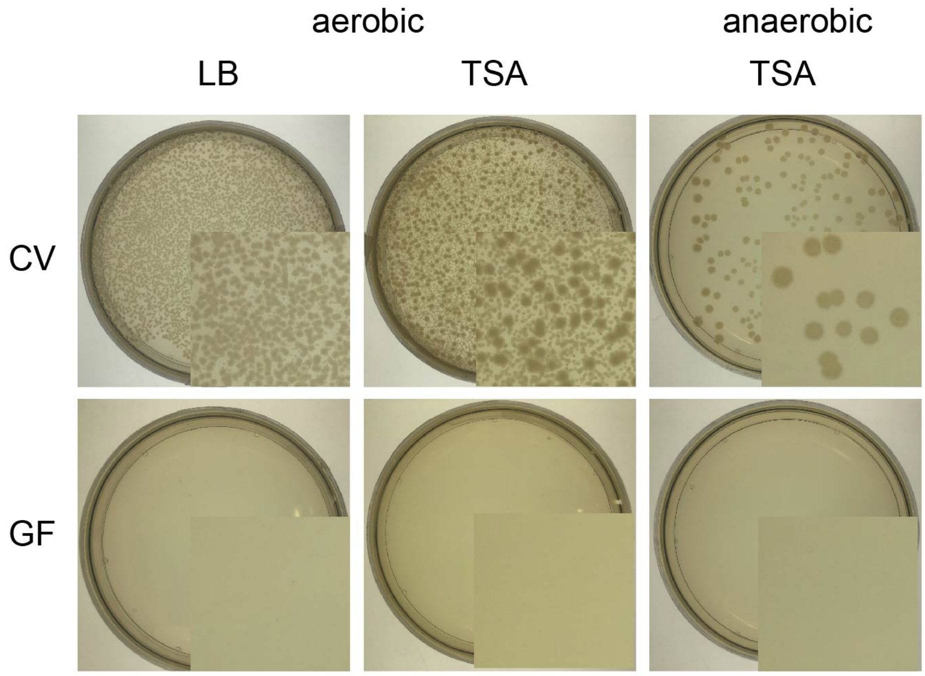

Figure S3

Germ-free condition of larval culture. Embryonic culture medium (ECM) after larval culture in conventional (CV) or germ-free (GF) conditions were spread to LB or TSA agar plates for culture in aerobic or anaerobic conditions. Enlarged images are also shown.

Figure S4

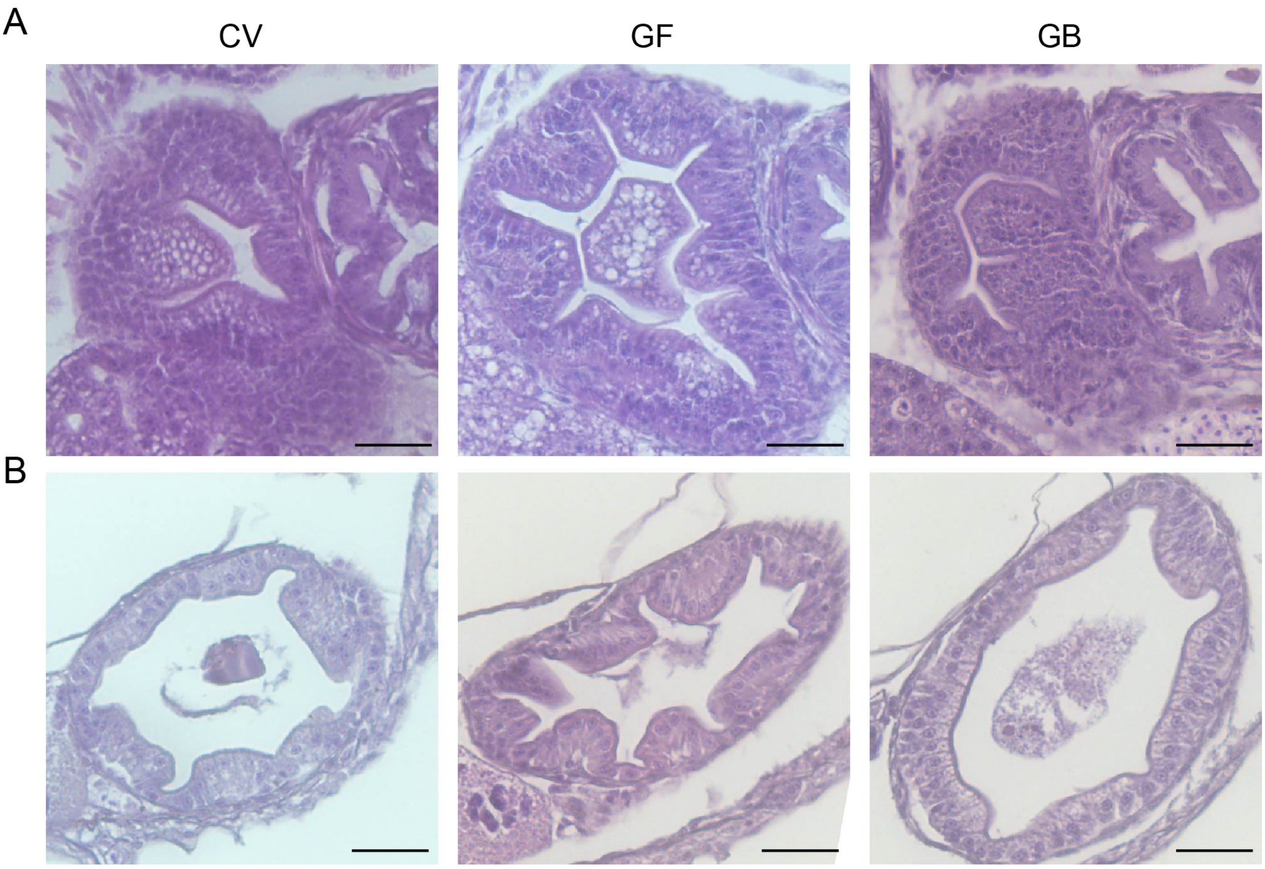

Figure S4

No apparent structural changes in the anterior and posterior intestinal epithelium. Coronal sections of anterior (A) and posterior (B) intestines after larval culture in conventional (CV), germ-free (GF), and gnotobiotic (GB) conditions. Scale bars; 50 μm.

Table S1 Oligos used for this study

Gene	Ensembl gene ID	Forward primer		Reverse primer		Comment
**Primers for qPCR**						
actb	ENSORLG00000013676	b-actin-F1	CCCCACCCAAAGTTTAG	b-actin-R1	CAACGATGGAGGGAAAGACA	
il2rg	ENSORLG00000001795	il2rg-F22	CCTGCTTTCTGTCTGCCACG	il2rg-R41	TTCACCACCAAACAGTCCAC	
cd79a	ENSORLG00000026911	cd79a-F1	TACATGCAGGTCTATGTGCC	cd79a-R1	CACAGAAACAGGAGGATTCC	
ebf1	ENSORLG00000002342	ebf1-F1	GAAGGCCAAGACAAGAACCC	ebf1-R1	ACAGGGTCTGAGGGTGTCTC	
cd22l	ENSORLG00000026274	cd22L-F1	TTGACGGCAGCAGCAACAGG	cd22L-R1	CCTTCAAAGGCACAGATGCG	
cd8a	ENSORLG00000008514	cd8a-F1	CTCTTGTGTTCTACCTGCAAG	cd8a-R2	GAACCAGAAGACCACGGTCC	
cd4-2	ENSORLG00000012768	cd4-2-F2	GGCTTCATCTTGTCTCAGTTG	cd4-2-R4	CATCAGCCACTTCACTGTAG	
mpx	ENSORLG00000009349	mpx-F22	GGATGCCAACAACAACGAGC	mpx-R21	AGAGGGTCAATTCCACCCTC	
nkl.1	ENSORLG00000022450	NKL.1-F11	CGTCTTGGTGATATCAGTCTG	NKL.1-R12	CATCCACTTACACCCCCAGC	
fasn	ENSORLG00000013515	fasn-F1	CATTCCTTGCTTTGGTCTCC	fasn-R1	CAAACGAGTAGCCAGCGATC	
apoa4a	ENSORLG00000009193	apoa4-F1	TTGGCCAGGACCTGAACTCC	apoa4-R1	GTGATGCGGCTGATCACATC	
gnr	ENSORLG00000004055	gnr-F1	CAGCTGCCCTCATTTCACAC	gnr-R1	GCGGATGACTCCATGTTCAG	
epx	ENSORLG00000009297	epx-F2	CCAACCAGCTCTATCCAGAC	epx-R1	ACAGGGTCAATTCCACCCTC	
hamp	ENSORLG00000028583	hamp-F1	AGTGACCTCATGGAAGACGC	hamp-R2	CACATCCACTCATCCCACAG	
hp	ENSORLG00000028170	hp-F1	GTGCAGCATTAGTCTTCCTG	hp-R2	CAGTGTTCCCCCAACCATTC	
c1qc	ENSORLG00000026388	C1q-F1	AGAACTGATGAGTAAAGCACC	C1q-R2	TGTTGGTGCTGAAGGGTCCG	
mrc1	ENSORLG00000017901	mrc1-F1	ACATGTACAGTTGGCTGGAC	mrc1-R1	CTCTCTGGCCTCATACCATG	
**Primers for RT-PCR**						
		igV$_H$116-F1	AGCCAGCCTCTGTTAGTCTG	igCm-R1	GAAATCAACGGCAAGACAGC	1st PCR
		igV$_H$116-F2	TGACCATCACCTGTCAGGTC	igCm-R2	AAGAGTGACCGTGTCTCTAC	2nd PCR
		igV$_H$37-F1	ACAGACTGACCTGTTCACCC	igCm-R1	GAAATCAACGGCAAGACAGC	1st PCR
		igV$_H$37-F2	TTGATTTCAGCAGCTATGCC	igCm-R2	AAGAGTGACCGTGTCTCTAC	2nd PCR
		tcrVb38-F1	GACAGCTGCCTGGAAGAACC	tcrCb2-R1	GTCTGAGGTCCACTCCTCAG	1st PCR
		tcrVb38-F2	GAGCAGATTGTGTGCACAAC	tcrCb2-R2	TCAGCCGGCTGGTGATTCTG	2nd PCR
		tcrVb51-F1	GGACATGCATTTCTCACCAC	tcrCb2-R1	GTCTGAGGTCCACTCCTCAG	1st PCR
		tcrVb51-F2	CCACTCCAAACTATGAGGAG	tcrCb2-R2	TCAGCCGGCTGGTGATTCTG	2nd PCR
**Primers for genotyping of CRISPR mutant**						
il2rg		il2rg-F32	ATGTGCAGAGTACTTGCCAG	il2rg-R32	CAGCACGTGCTCACCTTTCG	
**Primers for bacterial 16S rRNA gene amplification for direct sequencing**						
		16SrRNA 27F	AGAGTTTGATCCTGGCTCAG	16SrRNA 1491R	GGTTACCTTGTTACGACTT	
**Primers for bacterial 16S rRNA gene sequencing**						
				16SrRNA 907R	CCGTCAATTCCTTTRAGTTT	
**Primers for bacterial 16S rRNA gene amplification for Illumina sequencing**						
		16S_Amplicon_PCR_F	TCGTCGGCAGCGTCAGATGTGTATAAGAGACAGCCTACGGGNGGCWGCAG	16S_Amplicon_PCR_R	GTCTCGTGGGCTCGGAGATGTGTATAAGAGACAGGACTACHVGGGTATCTAATCC	
**sgRNA for genome editing**						
il2rg			CTGGCTGTAACCGACCATAC			

Table S2 Reproductivity of Aeromonadaceae enrichment in *il2rg* mutant intestines at 2.5 and 3 months post fertilization (M)

Age	WT		*il2rg* mutant		p value
	n	Aeromonadaceae composition (%), mean±SEM	n	Aeromonadaceae composition (%), mean±SEM	
**2 M**					
exp. 1	3	25.5±4.0	3	34.3±2.2	0.12
exp. 2	3	22.0±3.2	3	20.6±5.5	0.84
exp. 3	3	23.7±2.4	3	30.7±6.2	0.35
**2.5 M**					
exp. 1	3	14.0±1.5	3	48.2±4.0	0.0013
exp. 2	3	7.7±1.5	3	50.0±17.7	0.076
**3 M**					
exp. 1	5	35.4±13.2	4	81.3±5.1	0.022
exp. 2	3	9.4±1.6	1	72.7	-

Table S3 List of genes of decreased expression in germ-free larvae

gene_id	Product_Name	logFC	logCPM	LR	PValue	FDR	CV_1	CV_2	CV_3	GF_1	GF_2	GF_3
LOC101175226	DCN1-like protein 1	-9.32358449	6.011134556	48.36164246	3.54E-12	3.22E-08	125.154	86.496	22.397	0	0	0
LOC101167307	eosinophil peroxidase	-8.981717	5.861772651	19.00499979	1.30E-05	0.007171491	185.074	20.746	1.58	0.052	0	0
LOC111948971	hepcidin-like	-8.60598801	5.338044335	51.63984048	6.67E-13	1.21E-08	21.63	78.68	45.581	0	0	0
LOC105353613	NA	-8.56708108	5.299658224	15.78925011	7.08E-05	0.029948807	103.813	0	36.352	0	0	0
tbt-bp1	NA	-8.48076356	5.220037423	31.82506236	1.69E-08	3.83E-05	15.545	97.983	18.021	0	0	0
LOC101170304	nephronectin	-8.26203069	5.020138127	42.26084912	7.99E-11	4.83E-07	38.527	59.433	14.74	0	0	0
LOC101159074	complement C1q subcomponent subunit C	-7.87970906	4.675205218	29.19908499	6.53E-08	0.000118551	33.577	46.179	6.075	0	0	0
LOC105355191	uncharacterized LOC105355191	-7.78379001	4.595840526	15.00779697	0.000107068	0.038721599	49.175	0	33.437	0	0	0
LOC101173961	transmembrane protein 120A	-7.40456874	4.271737114	14.22464616	0.000162232	0.046936144	0	37.346	26.641	0	0	0
LOC105354882	uncharacterized LOC105354882	-7.22372009	4.116875348	37.69407334	8.28E-10	3.76E-06	27.133	14.213	13.943	0	0	0
LOC105355511	zinc finger protein 121-like	-7.0371073	3.963363989	32.34841136	1.29E-08	3.34E-05	14.986	24.107	9.295	0	0	0
LOC101172228	hepcidin	-6.99378397	3.924040931	19.65201659	9.29E-06	0.006245983	9.893	33.009	3.529	0	0	0
LOC105356343	alpha-tectorin-like	-6.50540731	3.551444918	29.35884339	6.01E-08	0.000118551	12.2	6.6	15.194	0	0	0
ppil1	peptidylprolyl isomerase like 1	-6.42485901	3.489529723	20.26226817	6.75E-06	0.005106695	5.11	19.645	6.878	0	0	0
LOC101167073	NEDD8	-6.36538101	3.446466588	23.74241015	1.10E-06	0.001052136	9.574	15.075	5.605	0	0	0
LOC101173030	gap junction Cx32.2 protein	-6.3316346	3.416646403	14.82074521	0.000118228	0.039742049	6.798	20.591	1.797	0	0	0
rfc5	replication factor C subunit 5	-6.13410066	3.283642664	23.27729875	1.40E-06	0.001272869	12.429	5.656	7.832	0	0	0
LOC101167094	myelin-oligodendrocyte glycoprotein-like	-5.95717771	3.16450859	24.11373087	9.08E-07	0.000970159	8.693	8.479	5.689	0	0	0
LOC101156852	uncharacterized LOC101156852	-5.89285626	3.120181395	26.50077296	2.63E-07	0.000434712	6.184	7.378	8.436	0	0	0
LOC101168243	forkhead box protein F2	-5.85146106	3.088568298	14.93803666	0.0001111	0.038721599	8.946	10.118	1.812	0	0	0
LOC101170257	haptoglobin	-5.68309078	2.984176541	15.86108672	6.82E-05	0.029948807	5.796	10.09	2.796	0	0	0
LOC101174703	ependymin-2	-5.45856468	3.254614897	23.88118009	1.02E-06	0.001033339	9.23	9.395	6.285	0	0.201	0
LOC101161339	regulator of G-protein signaling 20	-5.3940309	2.813192407	16.62870811	4.55E-05	0.020628718	2.91	7.197	5.339	0	0	0
mrpl58	mitochondrial ribosomal protein L58	-5.32141815	3.452637617	19.85529582	8.35E-06	0.006021435	6.228	16.737	7.215	0	0.392	0
stoml1	stomatin like 1	-5.29691253	2.758091271	14.90506746	0.000113059	0.038721599	7.313	3.862	3.115	0	0	0
LOC101156901	uncharacterized LOC101156901	-5.14237765	2.675784403	15.78464796	7.10E-05	0.029948807	5.465	4.203	3.198	0	0	0
LOC101156812	cell death activator CIDE-3	-5.10776098	2.657463535	15.48144793	8.33E-05	0.031508477	5.371	4.043	3.14	0	0	0
LOC111946765	uncharacterized LOC111946765	-5.06279889	2.632044557	14.91719563	0.000112334	0.038721599	2.664	5.449	4.081	0	0	0
LOC101171668	UNC93-like protein MFSD11	-4.90200199	4.303639215	20.86632758	4.92E-06	0.003886708	6.345	32.568	24.838	0	1.754	0
LOC101158478	prostaglandin reductase 1	-4.84548631	4.138782997	32.59603699	1.13E-08	3.34E-05	23.922	15.946	15.042	0	1.554	0
LOC105357389	IgGFc-binding protein	-4.02421243	4.641851699	25.40439349	4.65E-07	0.000602722	34.035	23.086	23.388	0	4.628	0
LOC105355038	sperm acrosome membrane-associated protein 4	-3.96235137	3.042402427	14.53282185	0.000137739	0.041670575	8.846	5.666	4.642	0	0.881	0
LOC101169902	transcription factor IIIA-like	-3.76544657	3.314904228	19.31143136	1.11E-05	0.006950311	8.973	6.837	9.59	0	1.51	0
LOC101172742	girdin	-3.72371083	4.50303751	17.16966872	3.42E-05	0.01677104	32.911	20.298	17.695	0	5.09	0
tfpt	TCF3 fusion partner	-3.62953685	3.02443724	14.32332967	0.000153945	0.045810041	4.19	7.594	6.871	0	1.154	0
LOC105354256	uncharacterized LOC105354256	-3.62397036	7.188711321	19.44136752	1.04E-05	0.006725033	94.754	158.012	273.644	13.352	27.141	1.531
LOC101163523	macrophage mannose receptor 1	-3.36350673	3.570135313	19.12670366	1.22E-05	0.007152836	12.289	10.576	8.648	1.08	1.646	0
LOC101166533	IgGFc-binding protein	-3.3094889	7.669759096	22.74056616	1.85E-06	0.001602667	383.109	184.982	131.438	26.279	37.956	7.851
LOC101173156	nucleoredoxin-like protein 2	-3.23965344	3.971893791	14.71257685	0.000125208	0.039821776	17.807	14.138	12.733	0	4.425	0
LOC101159706	band 3 anion exchange protein-like	-3.21529499	3.956007356	17.1731307	3.41E-05	0.01677104	18.371	16.082	9.323	2.792	1.636	0
LOC101168876	alpha-1,3-mannosyl-glycoprotein 4-beta-N-acetylglucosaminyltransferase B	-3.18503932	4.00419342	24.1127055	9.09E-07	0.000970159	14.471	14.009	17.657	2.923	1.791	0
LOC101174227	ependymin-2-like	-2.90392512	5.221185851	25.91399764	3.57E-07	0.000539978	56.89	26.323	34.298	5.144	5.502	4.751
LOC101174464	ependymin-2-like	-2.74690928	6.087384219	25.74930919	3.89E-07	0.000542837	106.381	52.084	60.719	7.271	10.481	14.493
LOC101161170	apolipoprotein A-IV	-2.7413894	8.488029213	19.06869056	1.26E-05	0.007152836	690.257	131.235	381.62	55.16	71.718	53.154
LOC105354936	von Willebrand factor A domain-containing protein 7	-2.69009925	8.008301075	16.7505009	4.26E-05	0.019842002	526.242	145.454	174.588	48.694	60.918	24.048
avl9	AVL9 cell migration associated	-2.674329	4.3370181	19.11292961	1.23E-05	0.007152836	21.662	16.805	19.699	5.285	3.058	0.457
fasn	fatty acid synthase	-2.64866674	4.573747079	15.71387906	7.37E-05	0.029948807	36.905	17.146	15.263	3.957	3.518	3.346
LOC101168633	granulins	-2.582812	4.174099521	17.28009182	3.23E-05	0.01672829	17.016	16.341	17.101	4.071	4.043	0
LOC101155030	NA	-2.53156492	5.291729304	14.76434484	0.000121817	0.039821776	44.545	57.075	16.7	4.515	8.822	7.074
gb-3	NA	-2.3849761	5.081418903	15.14277692	9.97E-05	0.036925956	43.031	40.359	16.165	6.565	7.511	4.947
gatm	glycine amidinotransferase	-2.33878603	4.84827399	14.65001031	0.000129434	0.039821776	38.933	22.04	22.489	3.315	3.724	9.067
LOC105357250	uncharacterized LOC105357250	-2.30025924	4.999298291	14.71958627	0.000124744	0.039821776	48.007	16.796	28.845	5.828	6.542	6.33
LOC101167806	glutathione S-transferase A	-2.29320302	4.242554051	14.2169002	0.000162901	0.046936144	21.072	17.739	12.47	5.144	2.559	2.503

Table S4 List of genes of increased expression in germ-free larvae

gene_id	Product_Name	logFC	logCPM	LR	PValue	FDR	CV_1	CV_2	CV_3	GF_1	GF_2	GF_3
mitd1	microtubule interacting and trafficking domain containing 1	8.065862567	4.8409988	15.65789835	7.59E-05	0.029948807	0	0	0	0	44.182	53.996
LOC101173858	alpha-1,3-mannosyl-glycoprotein 4-beta-N-acetylglucosaminyltransferase C-l	7.649934391	4.478611971	34.51488152	4.23E-09	1.54E-05	0	0	0	46.011	12.15	16.993
LOC101167208	monocarboxylate transporter 2	6.894002675	3.844661454	24.7471572	6.54E-07	0.000791005	0	0	0	8.294	8.232	26.771
LOC105354193	myoD family inhibitor domain-containing protein	5.525548427	2.888163615	17.52796147	2.83E-05	0.015114919	0	0	0	8.52	3.622	4.727
LOC105353825	tripartite motif-containing protein 16-like	3.34065289	3.463793105	15.65946127	7.58E-05	0.029948807	0	1.239	1.261	11.462	11.845	5.367
cubn	cubilin	2.556143986	5.266791496	21.08266346	4.40E-06	0.003629492	2.749	4.675	12.792	47.26	33.299	37.636
